# Plants of the Genus Zingiber: A Review of Their Ethnomedicine, Phytochemistry and Pharmacology

**DOI:** 10.3390/molecules27092826

**Published:** 2022-04-29

**Authors:** Miao Deng, Xuan Yun, Shurui Ren, Zhixing Qing, Fenglian Luo

**Affiliations:** 1 College of Food Science and Technology, Hunan Agricultural University, Changsha 410128, China; dengmiao1999@163.com (M.D.); zsb@hunau.net (S.R.); 2 Hunan Co-Innovation Center for Utilization of Botanical Functional Ingredients, College of Veterinary Medicine, Hunan Agricultural University, Changsha 410128, China; yunxuan0416@163.com

**Keywords:** genus *Zingiber*, phytochemistry, pharmacology, ethnomedicine, gingerols, *Zingiberaceae*

## Abstract

Plant of the genus *Zingiber* (*Zingiberaceae*) have primarily distributed in subtropical and tropical Asia, South America and Africa. The species of this genus have been widely used as food and in folk with a long history for treating various diseases. Reports related to the phytochemistry and phytochemistry of *Zingiber* species are numerous, but articles on the summary of the genus *Zingiber* remain scarce. This review aims at presenting comprehensive information about the genus *Zingiber* and providing a reference for the future application by systematically reviewing the literature from 1981 to 2020. Currently, a total of 447 phytochemical constituents have been isolated and identified from this genus, in which volatile oils, diarylheptanoids, gingerols, flavonoids and terpenoids are the major components. Gingerols, which are the main functional components, are the spicy and aromatic ingredients in the *Zingiber* species. Extracts and single compounds from *Zingiber* plants have been discovered to possess numerous biological functions, such as anti-inflammatory, anticancer, antimicrobial, larvicidal, antioxidant and hypoglycemic activities. This review provides new insights into the ethnomedicine, phytochemistry and pharmacology of the genus *Zingiber* and brings to the forefront key findings on the functional components of this genus in food and pharmaceutical industries.

## 1. Introduction

The genus *Zingiber* is the third largest of the family *Zingiberaceae*, whose members are mostly edible and medical plants [1]. It comprises 141 species, of which 12 species are native to China, southwest China in particular [2]. The plants of this genus are mostly perennial herbs with a fibrous rhizome, erectedg stem, and aromatic odor. The roots of *Zingiber* plants are mainly used for food and medicine, and the stems, leaves and roots are also used for extracting aromatic oils. Many types of chemical compounds of *Zingiber* have been discovered in current studies, such as volatile oils, organic acid, sterides, flavonoids, diarylheptanoids, gingerols and terpenoids. Although numerous chemical constituents of the genus *Zingiber* have been reported, including some well-known compounds named 6-gingerol (**325**), zerumbone (**1**) and curcumin (**294**), a systematic summary of the chemical constituents of this genus was rarely reported.

**Figure 1:**
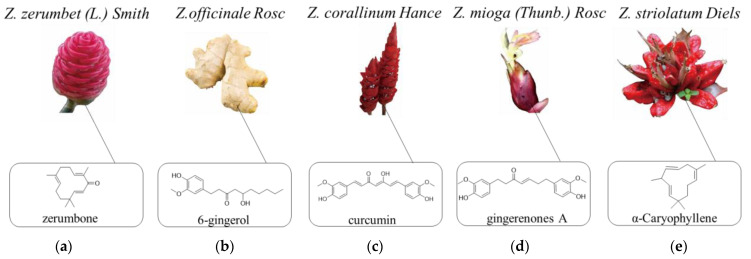
The characteristics of some *Zingiber* plants. ((**a**) *Zingiber zerumbet* (L.) *Smith*; (**b**) *Zingiber officinale Rosc*; (**c**) *Zingiber corallinum Hance*; (**d**) *Zingiber mioga (Thunb.) Rosc*; (**e**) *Zingiber striolatum Diels*).

As traditional medical and edible herbs, numerous studies have focused on five plants of the genus *Zingiber* (*Zingiber zerumbet* (L.) *Smith* (Figure 1a), *Zingiber officinale Rosc* (ginger) (Figure 1b), *Zingiber corallinum Hance* (Figure 1c), *Zingiber mioga (Thunb.) Rosc* (Figure 1d) and *Zingiber striolatum Diels* (Figure 1e). Modern pharmacological studies demonstrated that they exhibit extensive biological activities, such as antimicrobial, larvicidal, antioxidant, anti-obesity, anti-inflammatory, hypoglycemic, neuroprotective, cardiovascular protective and anti-tumor effects. According to their usage in history, *Zingiber* plants have been used to treat various symptoms and diseases, including nausea, vomiting, cough, common cold, and headache, relieving joint pain and menstrual cramp, and preventing stomach ulcers, neurodegenerative diseases, eye inflammation, cardiovascular diseases, diuretic and respiratory disorders [3,4,5].

Despite scholars having conducted extensive research on this genus in recent years, reports on summaries of the genus *Zingiber* remain scarce. Most of the research is especially concentrated on the *Z. officinale*, which is abundant in China, and is distributed mainly in the southwest to southeast of China, especially in the Guangdong, Guangxi, Yunnan, Sichuan and Guizhou provinces [6]. However, the details on the other species of this genus are scanty. Therefore, in this review, the ethnomedicine, chemical compositions and pharmacological activities from the available research reports on the genus *Zingiber* were systematically summarized and presented.

## 2. Methods of Data Collection

### 2.1. Methods

All of the available information presented in this review, concerning the genus *Zingiber*, was gathered via the scientific database, including PubMed, Google Scholar, Web of Science and Chinese National Knowledge Infrastructure (CNKI) between 1981 and 2020. In addition, part of the information was obtained from some local books, PhD and master’s dissertations. Keywords, such as *Zingiber*, phytochemical composition, bioactivities of *Zingiber*, and *Zingiberaceae*, were used for the online search. The structures of the *Zingiber* chemical contents, which were mentioned in the articles, were obtained from theses, books, databases and other reliable sources.

### 2.2. Inclusion Criteria

**Figure 2:**
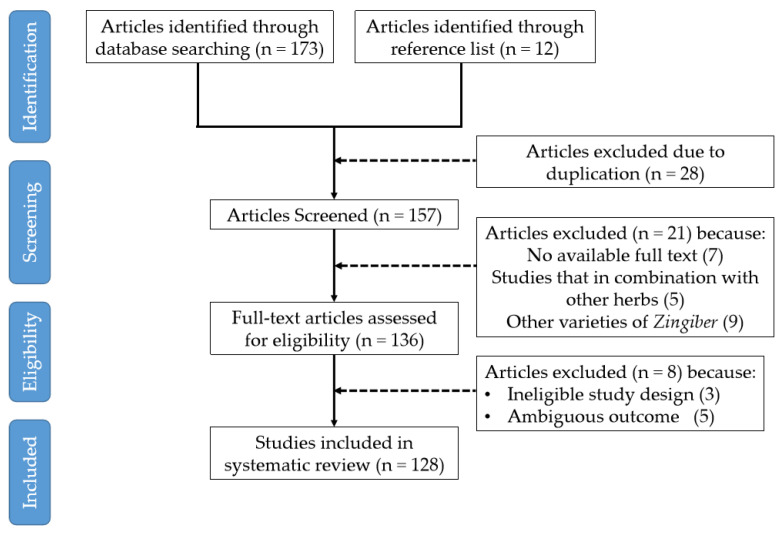
Flowchart of screening and choosing eligible articles.

Using the above-mentioned methods, we selected literature (1981–2020) concerning the ethnomedicine, phytochemistry and pharmacology of the genus *Zingiber*. Non-English articles were included if they included eligible study design and relevant outcomes. Considering the comprehensiveness of this review, all types of articles (books, research, reviews, clinical trials and meta-analysis) are included and ultimately 130 eligible articles as the result of the selection process. The details of screening and selecting eligible articles are available in Figure 2.

### 2.3. Exclusion Criteria

We excluded the articles whose study design is unreasonable or whose outcome is ambiguous.

## 3. Traditional Uses and Modern Applications

### 3.1. Traditional Medical Uses and Modern Applications of *Zingiber* Plants in China

Plants of the genus *Zingiber* have been used in China for centuries, which are recorded for treating cough, emesis, rhinobyon and typhia in the monographs of traditional Chinese medicine, such as Sheng Nong’s herbal classic and the Compendium of Materia Medica [7]. *Z. officinale* is the representative herbal medicine of the genus *Zingiber*, which is used as a common medication to relieve cough, cold, vomiting, diarrhea and abdominal pain in the traditional Chinese medicine system. Another species (*Z. zerumbet*) in this genus was recorded to have treatment effects of joint pain, stomachache, cold and dysmenorrhea. *Z. striolatum* has been documented to relieve diabetes and constipation in the Compendium of Materia Medica.

In addition to their traditional uses, plants of *Zingiber* can be used as prescription oral treatment for coronary heart disease, myocardial infarction, cardiovascular disease and enteritis [8,9]. *Z. officinale* has been found as a safe and well tolerated alternative to anti-emetic medications, which can decrease the severity and incidence of PONV (postoperative nausea and vomiting) [10]. Moreover, *Z. officinale*, as the best-known plant of the genus *Zingiber*, has different uses through different processing methods. Dried *Z. officinale* was used to cure enteritis, diarrhea and emesis, whereas baked *Z. officinale* was cured hemafecia, metrorrhagia and metrostaxis in the Chinese medicine system [11]. Ethyl-acetate extract of *Z. zerumbet* has a protective effect against ethanol-induced brain damage, which is mediated through its antioxidant properties [12]. With the unique fragrance and strong antimicrobial and insecticidal functions, *Z. corallinum Hance* has been widely used in the washing, fragrance and cosmetic industry.

### 3.2. Traditional Medical Uses and Modern Applications of *Zingiber* Plants in Other Countries

Plants of the genus *Zingiber* have played significant roles in the medicine system of many other countries. *Z. mioga* (Figure 1d) was used to ameliorate inflammation, rheumatic disorders and gastrointestinal discomforts in traditional Oriental medicines [13]. *Z. officinale* (Figure 1b) is employed as an important medicine for treating catarrh, rheumatism, nervous diseases, gingivitis toothache, asthma, stroke, constipation, and diabetes in the Ayurvedic and Tibb-Unani herbal medicines [14]. Moreover, *Z. officinale* has the effects of treating asthma, bronchitis, piles, eructation, constipation, ascites and relieving flatulence in the Indian medicine system [15,16]. The rhizome powder of *Z. zerumbet* (Figure 1a) mixed with ripe *Morinda citrifolia* is used for the treatment of severe pain in India. The fresh rhizome of *Z. zerumbet* is served as an anti-flatulent agent in Thailand. In addition, it also has a long history in the treatment of headache, toothache, ringworm, arthralgia, sprains and stomach-ache by Hawaiians [5].

Besides their medicinal uses, plants of *Zingiber* are the vital ingredient in the daily diets in other countries. The flower buds of *Z. mioga*, which have a pungent aroma, are used as spices, pickles and health supplements in Eastern Asia. As well, it is an excellent food ingredient for increasing the functionality and consumer acceptability of kimchi [17,18]. The leaves of *Z. mioga* can be used to wrap and preserve manjyu, which is a traditional Japanese confection [19].

## 4. Phytochemical Contents

**Figure 3:**
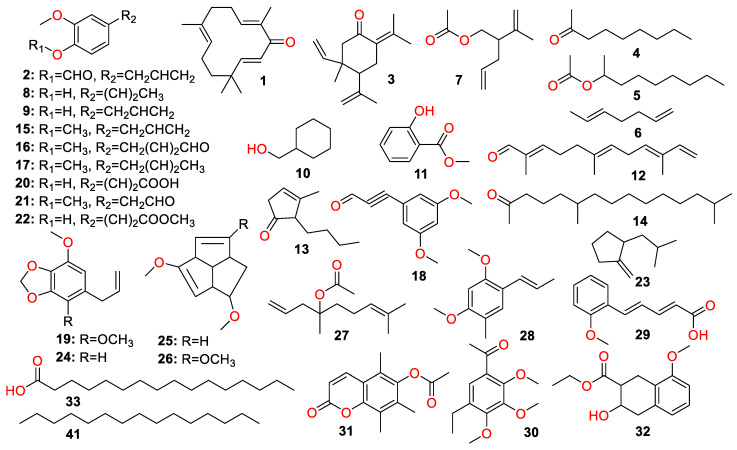

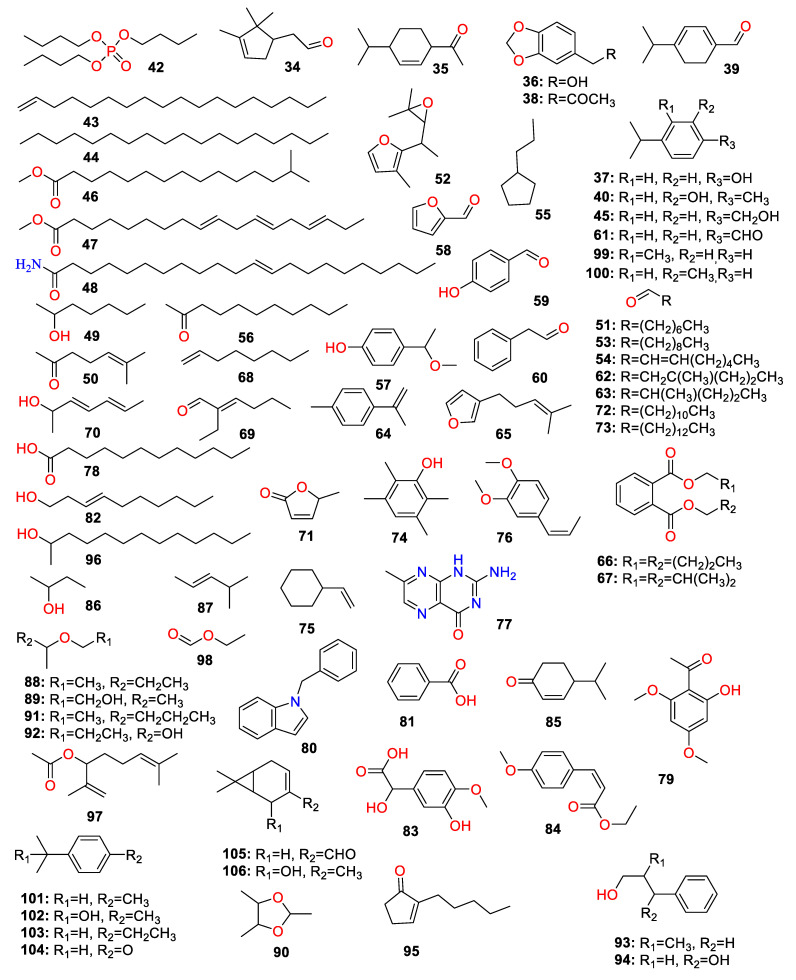
Chemical structures of compounds **1**–**106**.

**Figure 4:**
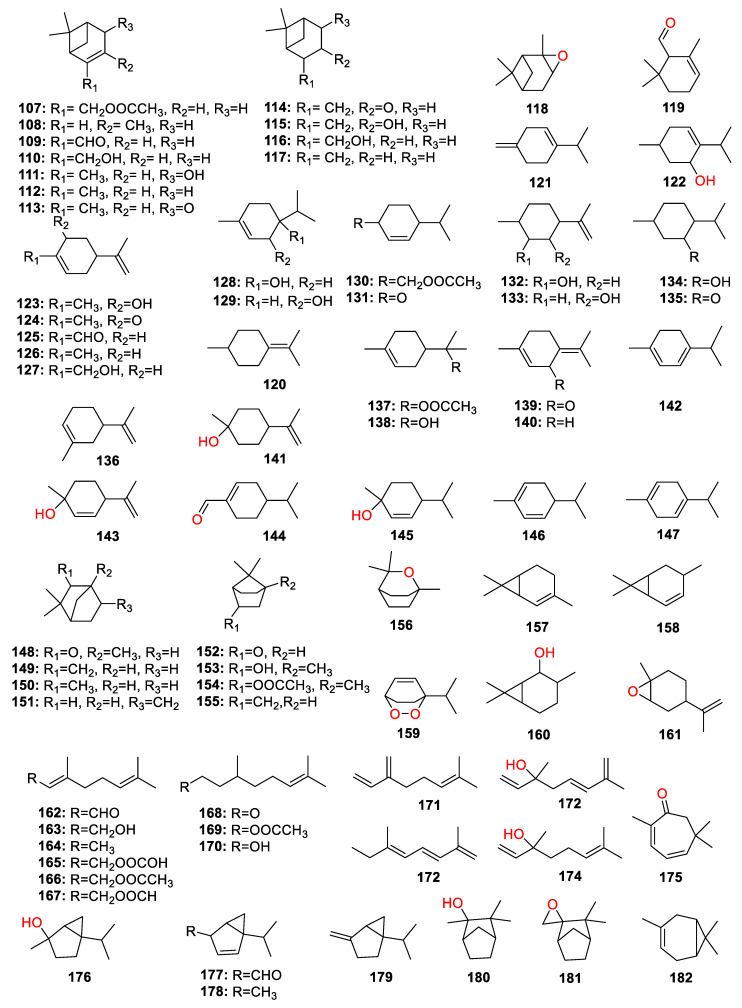
Chemical structures of monoterpenes (**107**–**182**).

**Figure 5:**
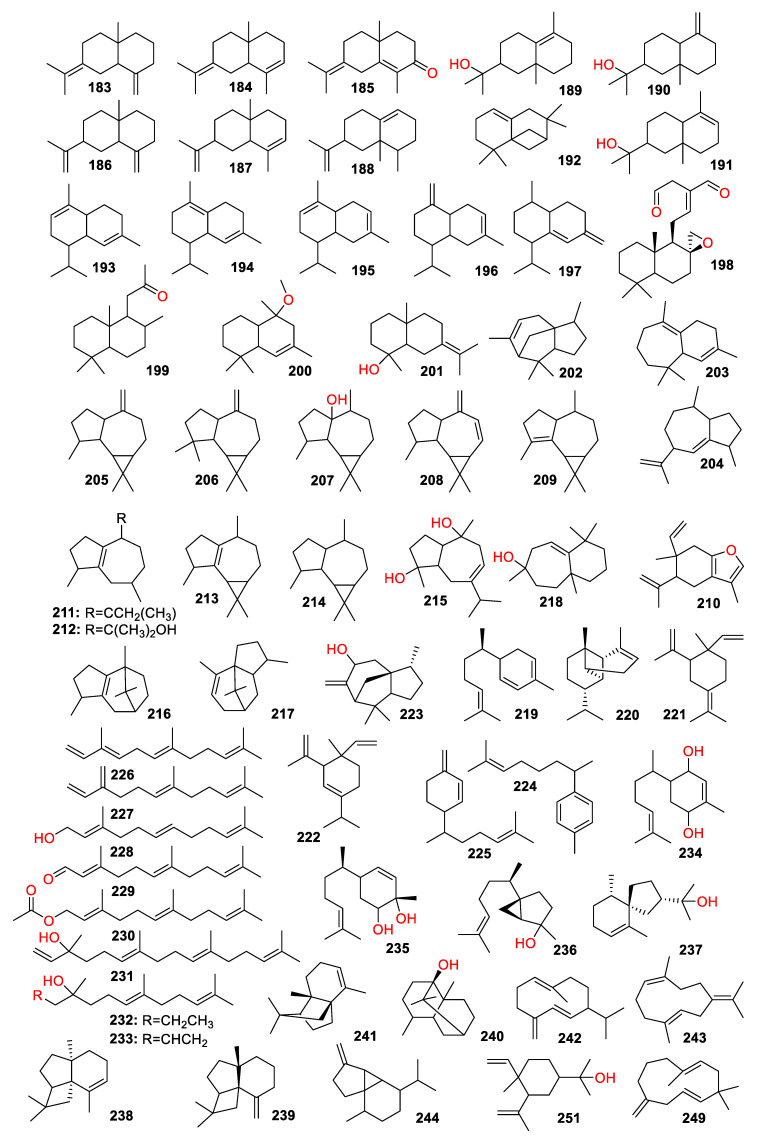

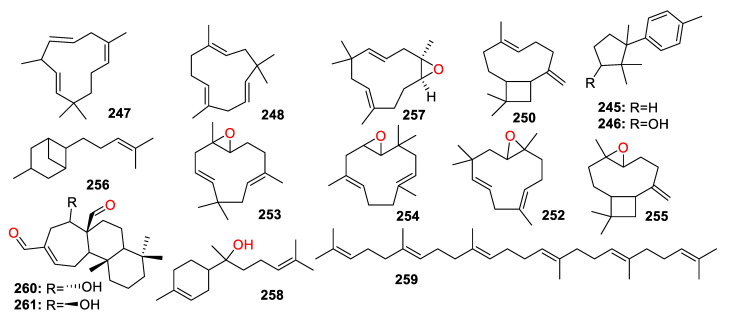
Chemical structures of sesquiterpenes, diterpenes and triterpenes (**183**–**261**).

**Figure 6:**
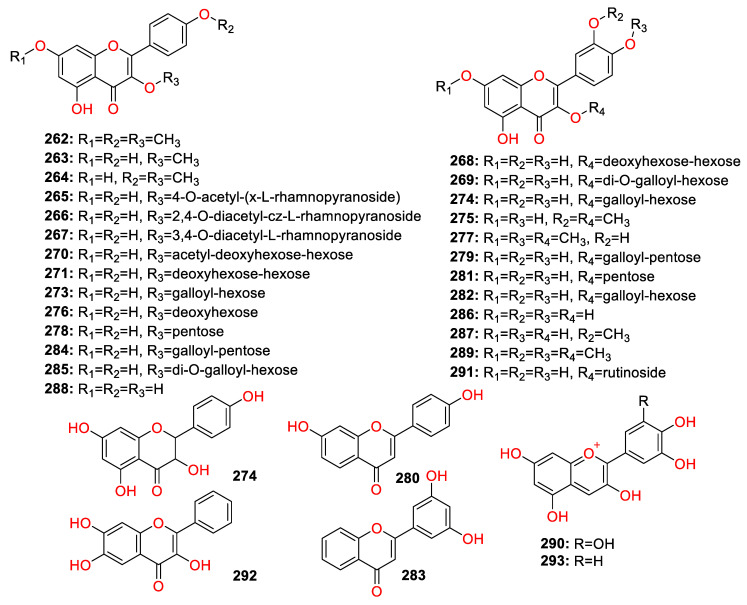
Chemical structures of the flavonoid compounds (**262**–**293**).

**Figure 7:**
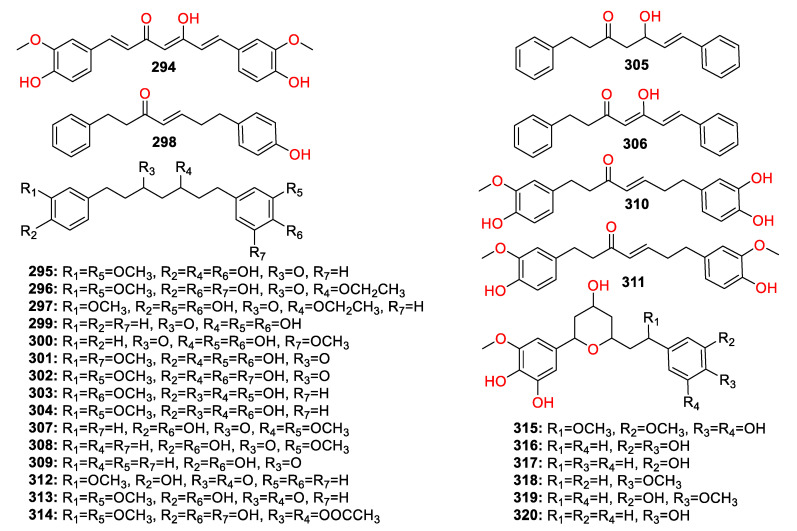
Chemical structures of the diphenylheptanes (**294**–**320**).

**Figure 8:**
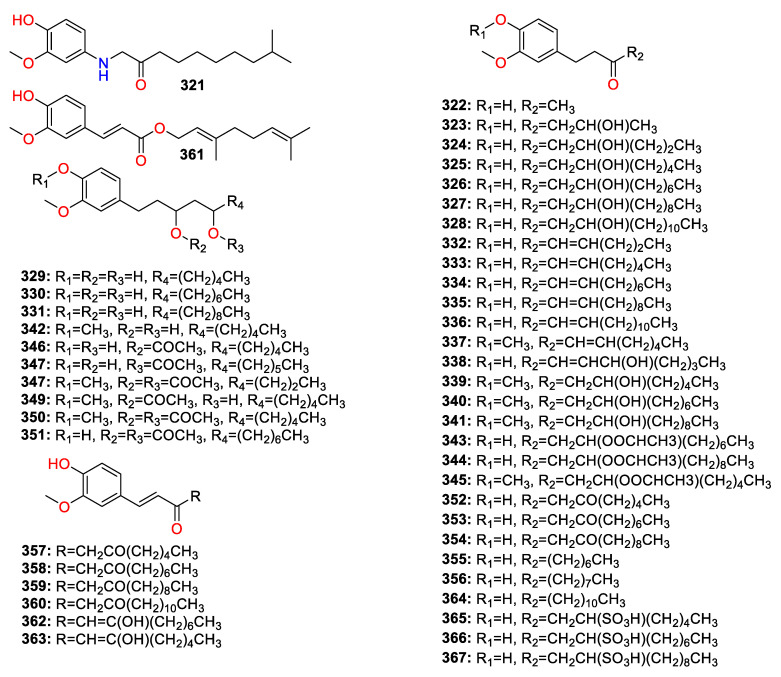
Chemical structures of the gingerol analogues (**321**–**367**).

**Figure 9:**
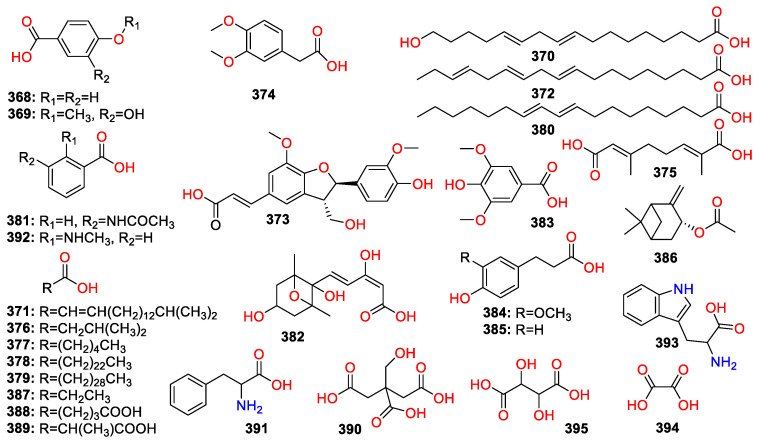
Chemical structures of the organic acids (**368**–**395**).

**Figure 10:**
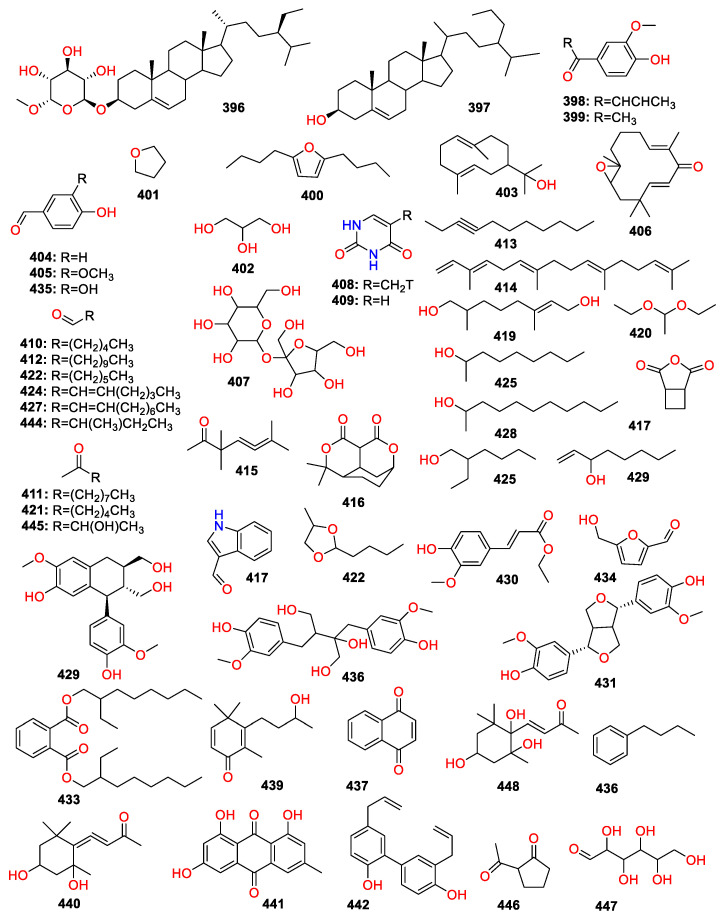
Chemical structures of the sterides and other compounds (**396**–**447**).

A total of 447 compounds have been isolated and identified from the genus *Zingiber*, which can be classified into seven categories, including volatile oils, terpenoids analogues, flavonoids, gingerol analogues, diarylheptanoids, organic acids and sterides. Those compounds and their origins have been summarized in Table S1 from Supplementary Materials, and their chemical structures have been described in Figure 3, Figure 4, Figure 5, Figure 6, Figure 7, Figure 8, Figure 9 and Figure 10.

### 4.1. Volatile Oils

Compounds **1**–**106** are the volatile oils that were isolated and identified from *Zingiber* plants (Figure 3). The volatile oils were extracted from the rhizome of *Zingiber* plants, which account for roughly 0.25~3.0%. The α-zingiberene (**219**) is the main component of the *Z. officinale* volatile oil [20,21]. Zerumbone (**1**) is the predominant component of *Z. zerumbet volatile* oils, whose analogs account for approximately 60.3% [22]. Palmitic acid (**33**) was identified as the highest content of the *Z. striolatum Diels* volatile oils, which account for about 30.5% [23].

### 4.2. Terpenoid Analogues

Compounds **107**–**261** are the terpenoid analogues. The odor of *Zingiber* plants can be attributed to the terpenoids’ compounds, which mainly consist of monoterpene (Figure 4) and sesquiterpene (Figure 5), such as ar-curcumene (**224**) and β-sesquiphellandrene (**225**) [24]. Three diterpene dialdehydes’ compounds, named galanal A (**260**), galanal B (**261**) and miogadial (**198**), were isolated from *Z. mioga*, in which the compound **198** was the main contribution to the pungent taste of the flower buds [25].

### 4.3. Flavonoids

A total of 32 flavonoids (compounds **262**–**293**) have been identified from the *Zingiber* plants (Figure 6). The flavonoids’ content of the *Z. mioga* bud was 0.48%, which was much higher than that of common vegetables (0.001–0.1%). Two anthocyanin compounds, delphinidin (**292**) and cyanidin (**293**), were considered as the potential hypoglycemic components in *Z. mioga* [26].

### 4.4. Diarylheptanoids

Twenty-seven diarylheptanoid compounds (**294**–**320**) were detected in the genus *Zingiber*, and curcumin (**294**) is the first diarylheptanoid compound that was isolated from this species (Figure 7). Diarylheptanoids are a class of compounds with 1,7-disubstituted phenyl groups and heptane framework, which can be subdivided into acyclic (2**94**–**314**) and cyclic diarylheptanoids. The aromatic rings of diarylheptanoids are easily hydroxylated and methoxylated, with the alkyl chains containing ketone, alkene, alcohol, and acetyl functionalities, and 1,5- or 3,6-oxy bridges (**315**–**320**).

### 4.5. Gingerol Analogues

Forty-five gingerol analogues compounds (**321**–**367**) were isolated and identified from the *Zingiber* species, which were the main metabolites of *Zingiber* plants (Figure 8). Gingerol analogues can be classified into six categories, including gingerol, shogaol, zingerone, paradol, gingerdione and gingerdiol, based on the difference of hydrocarbon chains [27]. Different gingerol analogues demonstrated similar biological activity, which may be influenced by the lengths of alkyl side chains. 6-gingerol (**325**) is the highest-content chemical component of gingerol analogues and the main pungency constituent of the fresh *Z. officinale* rhizome [28]. Due to their thermal instability, gingerols easily undergo dehydration reactions to form the corresponding shogaols (**332**–**338**), which are the non-volatile pungent ingredients in the *Zingiber* species. 6-paradol (**355**) is produced from 6-shogaol (**333**) by a biotransformation process and possesses similar biological activities to 6-shogaol (**333**) [29]. 6-gingesulfonic acid (**365**) accounts for 0.0013% of *Z. officinale*, with weak pungency and strong anti-ulcer activity [30].

### 4.6. Organic Acids

Compounds **368**–**395** were isolated and identified as organic acids in *Zingiber* plants (Figure 9). These ingredients can be divided into fatty acids (compound **390**) and aromatic acids (compound **368**). Oxalic and tartaric acids (**394**–**395**) are two major acids of *Z. officinale* rhizomes [31].

### 4.7. Sterides

Only two sterides compounds have been found in the genus *Zingiber*, including daucosterol (**396**) and 24-propylcholesterol (**397**) (Figure 10).

### 4.8. Others

Compounds **398**–**447** have been isolated and identified from the genus of *Zingiber*, including anthraquinones (**441**), furans (**400**–**401**), pyrimidine (**408**–**409**) and saccharides (**407** and **447**) (Figure 10).

## 5. Pharmacological Properties

*Zingiber* plants possess anti-inflammatory, anticancer, antimicrobial, larvicidal, antioxidant, hypoglycemic, analgesic, neuroprotective and cardiovascular protective effects. These pharmacological properties have been summarized in Table 1 in the following subsections.

### 5.1. Anti-Inflammatory Activity

The extracts of *Zingiber* plants have been used to treat inflammation. The extract of *Z. zerumbet* inhibited the inflammatory-mediated signaling pathways (NF-κB, MAPK and PI3K-Akt) in human macrophages via suppression of the release of pro-inflammatory mediators and the mRNA expression of pro-inflammatory factors [47]. The total essential oil of *Z. officinale* (28 mg/kg/d) prevents chronic joint inflammation, which may be attributed to the combined effects of both the aromatic essential oil and gingerols [48].

The anti-inflammatory effects of the single metabolites originated from the *Zingiber* plants have been well investigated. Zerumbone (**1**) has the effect of reducing the inflammatory response of acute lung injury (ALI) by inhibiting the Akt-NFκB activation [49]. 6-shogaol (**333**) and 6-dehydrogingerdione (**357**) display the inhibiting effect for binding between sICAM-1 (cell adhesion molecules) and VLA-4 (very late antigen) of the THP-1 (human monocytic cell line) cells, which are the main features of inflammation initiation. In addition, 10-gingerol (**327**), 6-shogaol (**333**), 8-shogaol (**334**) and 6-dehydrogingerdione (**357**) can inhibit direct binding between sICAM-1 and LFA-1 (lymphocyte function-associated antigen) of the THP-1 cells with IC_50_ value of 57.6, 27.1, 65.4 and 62.0 μM, respectively [32]. Moreover, the selective inhibition of pro-inflammatory cytokines for 6-gingerol (**325**) was also detected [33]. A few papers suggest that the strong anti-neuroinflammatory effects of *Z. officinale* are owing to 10-gingerol (**327**), not 6-gingerol (**325**). Those effects can be attributed to the inhibition of proinflammatory gene expression by blocking NF-κB activation, which can conduce to a reduced level of NO, IL-1β, IL-6 and TNF-α [50].

### 5.2. Anticancer Activity

The extracts and single metabolites originating from the *Zingiber* plants, especially in the essential oils, diarylheptanoids and gingerols, display significant anticancer activity. The essential oils from the fresh rhizome of *Z. zerumbet* displayed obvious cytotoxicity against K562 (human leukemia cell lines), PC-3 (human prostate cancer cell lines), A549 (human lung cancer cell lines) and MRC-5 (human fetal lung fibroblasts cell lines) cells with IC_50_ values range from 35.73 to 216.99 μM, which is stronger than that of the dry rhizome [22]. Zerumbone (**1**) is the main essential oil of *Z. zerumbet*, which exhibits a significant inhibitory effect on Hela (human cervical cancer cell lines), H460 (human lung cancer cell lines) and A549 cell lines with the IC_50_ value of 6.4 μg/mL, 15 and 25 μM, respectively [38]. β-myrcene (**171**) exhibited in vitro cytotoxicity on HepG2 (human liver cancer cell line), HCT116 (colon cancer cell line) and MCF7 (breast cancer cell line) cells with an IC_50_ value ranging from 2.51 to 3.28 μg/mL [35]. α-pinene (**160**) and β-pinene (**182**) also demonstrate strong cytotoxicity to SK-OV-3, HO-8910 and Bel-7402 cells (human tumor cell lines) [51]. Galanals (**260**–**261**) can induce the death of Jurkat human T-cell leukemia cells, which are characterized by DNA fragmentation and caspase-3 activation.

3,5-dioxo-1,7-bis(3-methoxy-4-hydroxy)-phenyl-heptane (**313**), which belongs to the diarylheptanoid type compound, displayed cytotoxic effect on BEL7404 (human lung cancer cell lines), CNE (human nasopharyngeal carcinoma cell lines), Hela and KB (human oral epithelial cancer cell lines) cells with IC_50_ values of 49.4 ± 3.4, 76.7 ± 5.4, 86.8 ± 10.5 and 27.7 ± 2.7 μM, respectively. Curcumin (**294**) and Gingerenone A (**311**) also exhibited a stronger inhibitory effect in those human cancer cell lines [36].

Gingerols, as the major active components of *Zingiber* plants, possess remarkable anti-cancer activities as well. The aliphatic chain and hydroxyl moieties that existed in 6-gingerol (**325**) and 6-shogaol (**333**) were proven to be responsible for the anti-cancer activities, and 6-gingerol (**325**) had the potential to bind with DNA and induced cell death by autophagy and caspase-3 mediated apoptosis [28]. The HepG2 cell lines can be induced by 6-gingerol (**325**) via autophagy and caspase-3 mediated apoptosis, and 6-gingerol (**325**) also demonstrates cytotoxic effect on K562 as well [34,39]. 10-gingerol (**327**), 6-shogaol (**333**) and 6-dehydrogingerdione (**357**) exhibited a cytotoxic effect on multiple cancer cells (BEL7404, Hela and KB cell lines), while 6-shogaol (**333**) has a stronger inhibitory effect than the other two compounds.

**Figure 11:**
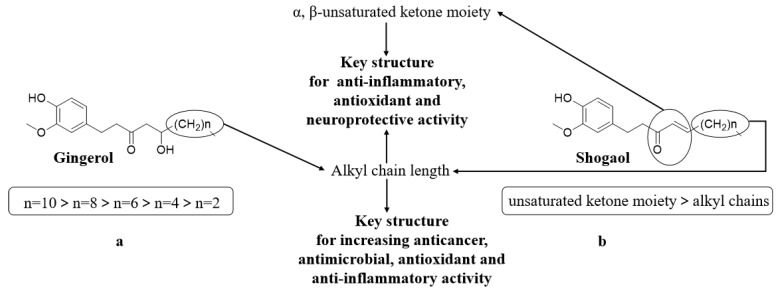
The structure–activity relationship of anticancer for gingerol (**a**) and shogaol (**b**).

The quantitative structure–activity relationship (QSAR) models found that the cytotoxicity was related to compound lipophilicity because it may increase the permeability of the cancer cell membrane [36]. This means that gingerols with longer unbranched alkyl side chains may have greater anticancer potential because of their increased lipophilicity (Figure 11a).

### 5.3. Antimicrobial Activity

The essential oils and gingerols originating from the *Zingiber* plants display significant antimicrobial activities and the details are available in Table S2 from Supplementary Materials. Essential oils of the *Z. officinale* rhizome have the significant effect of inhibiting the growth of both Gram-negative and Gram-positive bacteria [45]. Zerumbone (**1**) is the main antimicrobial ingredient of the *Z. zerumbet* essential oils, with a mid-to-high IC_50_ value against *staphylococcus aureus*, *bacillus subtilis*, *escherichia coli* and proteus vulgaris [52]. It also demonstrates an anti-virulence effect by inhibiting the biofilm formation and hyphal growth of *C.* *albicans* in a concentration-dependent manner, and it exhibited antimicrobial activity at the MIC (minimum inhibitory concentration) of 250 μg/mL against the *H. pylori* strain [53,54]. Three compounds named miogadial (**198**), galanal A (**260**) and galanal B (**261**) that isolated from *Z. mioga*, had antimicrobial activities against different strains of bacteria, yeasts and molds. However, miogadial (**198**) demonstrated stronger antimicrobial activity against Gram-positive bacteria and yeasts than compounds galanal A (**260**) and galanal B (**261**) [25]. The essential oil of *Z. corallinum Hance* also can inhibit the growth of numerous plant pathogenic fungi with a low concentration.

The majority of gingerol analogues displayed strong antimicrobial activity. For instance, 10-gingerol (**327**) has the stronger inhibitory effect on three periodontal bacteria than 12-gingerol (**328**), with a MIC range from 6 to 14 μg/mL and a MBC (minimum bactericidal concentration) range from 4 to 14 μg/mL. Bacteria of *Mycobacterium avium* and *Mycobacterium tuberculosis*, which may cause tuberculosis, can also be suppressed by 10-gingerol (**327**) as well. Furthermore, 10-gingerol (**327**) and 12-gingerol (**328**) from the *Z. officinale* rhizome have antibacterial activity against periodontal bacteria, with the MBC ranging from 4 to 20 μg/mL. The compound 6-gingerol (**325**) demonstrated a strong antimicrobial effect on *Helicobacter pylori*, with a MIC of 20 μg/mL. The structure–activity relationships (SARs) demonstrated that the shorter alkyl side chain of gingerols play a key role for their microbial inhibition effect (Figure 11a) [55].

### 5.4. Larvacidal Activity

The essential oils of *Zingiber* plants are proven to be an effective tool for mosquito larval control and the details are available in Table S3. The *Z. corallinum Hance* essential oil exhibited a stronger poison effect against the larval of *Aedes albopictus* and *Culex quinquefasciatus* than pupa [56]. Both methanol and dichloromethane extracts of *Z. zerumbet* rhizomes exhibited significant larvacidal effect. The dichloromethane extracts displayed more toxicity to the larvae of *Aedes aegypti* and *Anopheles nuneztovari* than the methanol extracts, which may be attributed to the essential oil. In addition, the gingerols were the vital insecticidal composition of the genus *Zingiber*. 4-gingerol (**324**) demonstrated strong larvicidal activity against *Aedes aegypti* and *Culex quinquefasciatus* [57]. β-sitosterol (**397**) was highly effective against *Aedes aegypti*, *Anopheles stephensi* and *C. quinquefasciatus*, with an LC_50_ value of 11.49, 3.58 and 26.67 ppm, respectively [58].

### 5.5. Antioxidant Activity

The polyphenols, flavonoids, gingerols and essential oils originating from the *Zingiber* plants demonstrated significant antioxidant activity. The polyphenols and flavonoids of *Z. mioga* account for approximately 0.5% and 4.6%, respectively, which were far higher than that in common vegetables (0.001~0.1%), manifesting its potential antioxidant properties. The ethanol extract of *Z. mioga*, compared to the water extract, exhibited stronger peroxyl radical scavenging linked antioxidant activity (0.53/TE 1 μM) with 2697.31 ± 118.25 mg/100 g of the total antioxidant capacity (TAC) [59]. The ethyl-acetate extract of *Z. zerumbet* at 400 mg/kg has a protective effect against ethanol-induced brain damage because of its antioxidant properties [11]. The antioxidant capacity of the methanolic extract of *Z. officinale* has been assessed with the DPPH assay (86.26 ± 0.97%), ABTS assay (91.04 ± 0.96%) and nitric oxide assay (86.72 ± 1.51%) [60].

The essential oils, such as zerumbone (**1**), display significant antioxidant power with a FRAP (Ferric-reducing antioxidant power) value of 58.3 ± 2.08, which is higher than that of ascorbic acid, by enhancing the cellular antioxidant pathway [53]. In addition, some gingerol analogues exhibited conspicuous antioxidant activity. 6-gingerol (**325**), 8-gingerol (**326**), 10-gingerol (**327**) and 6-shogaol (**333**) represented antioxidant effects with a IC_50_ value range from 8.05 to 26.3 μM against the DPPH radical. 10-gingerol (**327**) demonstrates a stronger quenching ability of DPPH radicals than curcumin (**294**), but a weaker quenching ability than quercetin (**286**). The highest antioxidant activity of 6-shogaol (**333**) can be attributed to the presence of unsaturated ketones moieties. The SAR demonstrated that the substituent groups and the length of the alkyl chain play a crucial role for their antioxidant effects and the presence of α; the β-unsaturated ketone moiety is predominant to that of the alkyl side chains’ length in exhibiting the antioxidant and anti-inflammatory properties (Figure 11b) [61,62].

### 5.6. Hypoglycemic Activity

The extracts and single metabolites originating from the *Zingiber* plants display significant hypoglycemic activity. The sucrase, maltase and α-amylase were significantly suppressed by the ethanol extract of *Z. mioga*. Moreover, the ethanol extract of *Z. mioga* exhibited it’s possibility of acting as an intestinal α-glucosidase inhibitor by using SD rat and db/db mice models [52]. The hypoglycemic activity of *Z. mioga* was considered to be the presence of the anthocyanin compound (**292**–**293**) [26]. The ethanol extract of *Z. striolatum* has a dose-dependent hypoglycemic effect on insulin-resistant HepG2 cells with low cytotoxicity [63]. A daily feeding of 200 mg/kg ethanolic extract of *Z. officinale* for 20 days can significantly decrease blood glucose [64]. In addition, it also inhibits LDL (low-density lipoprotein) oxidation [65] and HMG-COA (3-hydroxy-3-methylglutaryl coenzyme A) reductase and increases insulin release [66].

Gingerols exert their anti-diabetic effects primarily by activating AMPK (AMP-activated protein kinase), which regulates the glucose and lipid metabolism energy sensor. The steaming process would enhance the anti-diabetic potential of *Z. officinale* via increasing the content of 6-dehydrogingerdione (**357**), which could stimulate the insulin secretion by the closure of K_ATP_ (ATP-sensitive potassium channels) in pancreatic β-cells [67]. Furthermore, Gingerenone A (**311**) is equipped to sensitize the insulin receptor and increase glucose uptake by inhibiting the activity of p70 S6 kinase [68].

### 5.7. Prevention of Nausea and Vomiting Activity

*Z. officinale* has been used as an antiemetic in various traditional medicine systems for over 2000 years, and it remains considered as an alternative therapy for nausea and vomiting in modern medicine [69]. Doctors in Thailand used *Z. officinale* as a drug to prevent nausea and vomiting after laparoscopic surgery for gynecological outpatients [7]. Besides its medical application on postoperative nausea, *Z. officinale* was used for motion sickness and pregnancy-induced nausea and vomiting as well; those effects of reducing nausea and vomiting might be associated with a weak inhibitory effect of gingerols and shogaols on M3 and 5-HT3 receptors or exert their anti-emetic effect by acting on the 5-HT3 receptor ion-channel complex [70].

### 5.8. Others

Several other pharmacological activities of the components or extracts of the *Zingiber* species have been found in previous research, such as analgesic, anti-ulcer, neuroprotective and cardiovascular protection. The genus *Zingiber*, especially *Z. officinale*, possess remarkable analgesic activity, which could be due to their phytoconstituents binding to TRPA1 (ankyrin receptors) and TRPV1 (vanilloid receptors) ion channels [71]. The significant central and peripheral antinociceptive effects of the *Z. zerumbet* essential oil has been detected [72]. Zerumbone (**1**) displayed anti-hyperalgesic properties via suppressing the pain transmission from primary afferent neurons to the ascending tract and modulating pain impulses reaching the supraspinal regions [73].

An intraperitoneal injection of 25 mg/kg–50 mg/kg of 6-gingerol (**325**) into a rat can generate the inhibitory effect of the acetic acid-induced writhing response and formalin-induced licking time [74]. *Z. zerumbet* enhanced the protection of the ethanol-induced gastric ulcer by the effects of maintaining mucus integrity, antioxidant activity and HSP-70 induction [42]. Furthermore, *Z. officinale* powder can obviously improve the gastric mucosa injury caused by aspirin as well [75]. Moreover, 6-gingerol (**325**) was tested and has the ability of preventing the acrylonitrile-induced cerebral cortex lesion as well, with the increase in the brain immunohistochemical expression of caspases-9 and caspases-3. It would be a better fungible drug for the prevention of neurodegenerative diseases when compared to some synthetic drugs in [76]. The presence of a double bond and the linear chain of 6-shogaol (**333**) may enhance the neuroprotective effects of this compound (Figure 11b).

## 6. Conclusions

In this work, the phytochemical constituents and pharmacological effects of the *Zingiber* species was first reviewed systematically, based on the literature from 1981 to 2020. A total of 447 metabolites are included in this review, of which 34.78% are volatile oils, 23.70% are terpenoids, 6.96% are flavonoids, 5.87% are diarylheptanoids, 9.78% are gingerols analogues, 6.30% are organic acids and 12.61% are classified as other compounds. These compounds, including zerumbone (**1**), zingerone (**322**), curcumin (**294**) and gingerols (**321**–**367**), are considered the characteristic constituents of this genus. Gingerols are the main pungency components of the genus *Zingiber*, in which the 6-gingerol (**325**) accounts for more than 75% [7].

The anti-inflammatory, anticancer and antimicrobial effects are the main biological activities of the extract or single compound of these genus plants. As we all know, most plants of the genus *Zingiber* are medicine-food homology herbs. Therefore, the extract or single compound has a huge potential for the development of new food additives for their obvious biological activity. Some bioactive constituents of the *Zingiber* plants, such as 6-gingerol, 10-gingerol and 12-gingerol, displayed stronger antimicrobial activity, and are regarded as attractive targets in food contaminations management. In addition, the essential oil of *Z. corallinum Hance* holds great potential as an environmentally friendly pesticide, with a remarkable inhibitory effect of numerous plant pathogenic fungi. There is some evidence that *Zingiber* plants *(Z. officinale*, *Z. mioga* and *Z. striolatum*) may provide potential benefits on metabolic syndromes (obesity and type-2 diabetes). It is noteworthy that the ethanol extract demonstrates stronger hypoglycemic activity compared to the water extract in most animal studies. This may be due to ethanol extracting more flavonoid compounds, such as delphinidin (**292**) and cyanidin (**293**). As extensively used hypoglycemic drugs, such as acarbose, can cause side effects such as nausea, vomiting, gastrointestinal swelling and kidney function disorders, plants of the genus *Zingiber* could be used as a complementary or alternative medicine to diabetes therapy. *Z. officinale* has been used as an antiemetic for over 2000 years, which would be associated with gingerols and shogaols, and it remains considered as an alternative therapy for nausea and vomiting in motion sickness by the Committee on Herbal Medicinal Products (HMPC).

Despite possessing the significant pharmacological activity of some *Zingiber* constituents, the clinical applications are still rare, considering the uncertain safety of their consumption in humans. Furthermore, as products and studies related to the mechanisms underlying the pharmacological activity derived from those herbs remain scarce, more laboratory investigations and product developments are needed.

Structural modification by heating and dehydration, as well as enzyme reactions, may be helpful in enhancing the biological activities of *Zingiber* plants. Shogaols (**332**–**336**) are produced from gingerols (**323**–**327**) by heating, which are more effective in inhibiting the inflammatory mediators and ROS production and possess better thermostability as compared to gingerols (**323**–**327**). 6-paradol (**355**) is the non-pungent metabolite of 6-shogaol (**333**), which may avoid side effects such as gastric irritation. However, the structure–activity information related to *Zingiber* plants remains scarce, due to the variability of experimental outcomes. These metabolite contents were very low in *Zingiber* plants or difficult to isolate due to their structural similarity. Therefore, more extensive studies in this direction are needed for future clinical applications or for serving as adjuvants.

In conclusion, *Zingiber* plants are the herbs in homologous medicine and food that have been widely used in different countries for centuries. Our present paper provides comprehensive information on the traditional uses, phytochemistry and pharmacology of the genus *Zingiber*. We highlight the enormous potential of the *Zingiber* plants to serve as potent clinical drug candidates, in order to provide a scientific foundation for future research and application on this genus.

## Figures and Tables

**Table 1:** Pharmacological effects of *Zingiber* plants.

Pharmacological Effects	Details	Extracts/Compounds	Inhibitory Concentrations/Dose	References
**Anti-inflammatory activity**	Inhibition of the direct binding between intercellular adhesion molecules and lymphocyte function-associated antigen-1 of the THP-1 cells	10-gingerol	IC_50_: 57.6 μM	[32]
		8-shogaol	IC_50_: 65.4 μM	
	Inhibitory effect on direct binding between sVCAM-1 and VLA-4 of THP-1 cells	6-shogaol	IC_50_: 27.1 μM	
		Dehydro-6-gingerdione	IC_50_: 62.0 μM	
	Inhibition of the produc-tion of pro-inflammatory cytokines from LPS stim-ulated macrophages	6-gingerol	50 mg/kg	[33]
	Decreased ETBF-induced colitis via inhibition of NF-κB signaling	Zerumbone	MIC: 32–48 μg/mL	[34]
**Anti-cancer activity**	Cytotoxic effect on MRC-5 (human fetal lung fibroblasts cell linse)	*Z. zerumbet* fresh rhizome essential oil	IC_50_: 216.99 ± 8.27 μM for 24 h	[22]
		*Z. zerumbet* dry rhizome essential oil	IC_50_: 159.47 ± 9.34 μM for 24 h	
		Zerumbone	IC_50_: 117.96 ± 5.67 μM for 24 h	
	Cytotoxic effect on PC-3 (human prostate cancer cell lines)	*Z. zerumbet* fresh rhizome essential oil	IC_50_: 53.32 ± 1.34 μM for 24 h	
		*Z. zerumbet* dry rhizome essential oil	IC_50_: 77.45 ± 0.46 μM for 24 h	
		Zerumbone	IC_50_: 30.78 ± 1.31 μM for 24 h	
		*Z. striolatum* essential oil	IC_50_: 86.05 μM	[35]
		6-shogaol	IC_50_: 100.0 ± 13.1 μM	[36]
		6-dehydrogingerdione	IC_50_: 106.4 ± 12.5 μM	
		10-gingerol	IC_50_: 59.7 ± 8.2 μM	
		3,5-dioxo-1,7-bis(3-methoxy-4-hydroxy)-phenyl-heptane	IC_50_: 153.5 ± 13.8 μM	
		Gingerenone A	IC_50_: 114.3 ± 14.2 μM	
		3,5-diacetoxy-1-(3-methoxy-4,5-dihydroxy-phenyl)-7-(4-hydroxy-3-methoxyphenyl) heptane	IC_50_: 86.6 ± 7.5 μM	
		Curcumin	IC_50_**:** 16.5 ± 2.7 μM	
	Cytotoxic effect on K562 (human leukemia cell lines)	*Z. zerumbet* fresh rhizome essential oil	IC_50_: 35.73 ± 1.72 μM for 24 h	[22]
		*Z. zerumbet* dry rhizome essential oil	IC_50_: 41.79 ± 1.18 μM for 24 h	
		Zerumbone	IC_50_: 10.08 ± 0.61 μM	
		*Z. striolatum* essential oil	IC_50_: 29.67 μM	[35]
		6-gingerol	IC_50_: 22.86 μM	[37]
		Gingerenone A	IC_50_: 33.3 ± 5.1 μM	
		3,5-diacetoxy-1-(3-methoxy-4,5-dihydroxy-phenyl)-7-(4-hydroxy-3-methoxyphenyl) heptane	IC_50_: 39.6 ± 4.8 μM	
		1,5-epoxy-3-hydroxy-1-(3,4-hydroxy-5-methoxyphenyl)-7-(4-hydroxy-3-methoxyphenyl) heptane	IC_50_: 160.3 ± 14.1 μM	
		Citrylidenmalonsaeure	IC_50_: 119.8 ± 9.9 μM	
		Curcumin	IC_50_: 30.5 ± 5.3 μM	
	Cytotoxic effect on A-549 (human lung cancer cell lines)	*Z. zerumbet* fresh rhizome essential oil	IC_50_: 44.88 ± 1.21 μM for 24 h	[27]
		*Z. zerumbet* dry rhizome essential oil	IC_50_: 68.06 ± 1.09 μM for 24 h	
		Zerumbone	IC_50_: 25 μM	[38]
		*Z. striolatum* essential oil	IC_50_: 48.87 μM	[35]
		6-shogaol	IC50: 22.9 ± 2.1 μM	[36]
		6-dehydrogingerdione	IC50: 81.2 ± 9.6 μM	
		10-gingerol	IC50: 85.4 ± 10.2 μM	
		3,5-dioxo-1,7-bis(3-methoxy-4-hydroxy)-phenyl-heptane	IC_50_: 248 ± 17.9 μM	
		Gingerenone A	IC_50_: 44.5 ± 5.8 μM	
		3,5-diacetoxy-1-(3-methoxy-4,5-dihydroxy-phenyl)-7-(4-hydroxy-3-methoxyphenyl) heptane	IC_50_: 96.3 ± 7.8 μM	
		7-(3,4-dihydroxy-5-methoxyphenyl)-5-hydroxy-1-(4-hydroxy-3-methoxyphenyl) heptan-3-one	IC_50_: 83.6 ± 8.4 μM	
		1,5-epoxy-3-hydroxy-1-(3,4-hydroxy-5-methoxyphenyl)-7-(4-hydroxy-3-methoxyphenyl) heptane	IC_50_: 230.8 ± 17.9 μM	
		5-[4-hydroxy-6-(4-hydroxyphenethyl) tetrahydro-2H-pyran-2-yl]-3-methoxybenzene-1,2-diol	IC_50_: 212.0 ± 15.2 μM	
		Curcumin	IC_50_: 58.8 ± 9.4 μM	
	Cytotoxic effect on H-460 (human lung cancer cell lines)	Zerumbone	IC_50_: 15 μM	[38]
	Cytotoxic effect on BEL7404 (human lung cancer cell lines)	6-shogaol	IC_50_: 11.8 ± 2.6 μM	[36]
		6-dehydrogingerdione	IC_50_: 115.2 ± 13.7 μM	
		10-gingerol	IC_50_: 95.2 ± 12.2 μM	
		3,5-dioxo-1,7-bis(3-methoxy-4-hydroxy)-phenyl-heptane	IC_50_: 49.4 ± 3.4 μM	
		Gingerenone A	IC_50_: 9.0 ± 2.3 μM	
		3,5-diacetoxy-1-(3-methoxy-4,5-dihydroxy-phenyl)-7-(4-hydroxy-3-methoxyphenyl) heptane	IC_50_: 101.9 ± 13.1 μM	
		7-(3,4-dihydroxy-5-methoxyphenyl)-5-hydroxy-1-(4-hydroxy-3-methoxyphenyl) heptan-3-one	IC_50_: 180.6 ± 16.5 μM	
		1,5-epoxy-3-hydroxy-1-(3,4-hydroxy-5-methoxyphenyl)-7-(4-hydroxy-3-methoxyphenyl) heptane	IC_50_: 180.6 ± 12.5 μM	
		Curcumin	IC_50_: 38.2 ± 3.9 μM	
	Cytotoxic effect on CNE (human nasopharyngeal cancer cell lines)	6-shogaol	IC_50_: 43.8 ± 5.0 μM	
		6-dehydrogingerdione	IC_50_: 119.7 ± 7.9 μM	
		10-gingerol	IC_50_: 88.1 ± 7.3 μM	
		3,5-dioxo-1,7-bis(3-methoxy-4-hydroxy)-phenyl-heptane	IC_50_: 76.7 ± 5.4 μM	
		Gingerenone A	IC_50_: 27.7 ± 3.9 μM	
		3,5-diacetoxy-1-(3-methoxy-4,5-dihydroxy-phenyl)-7-(4-hydroxy-3-methoxyphenyl) heptane	IC_50_: 62.0 ± 10.7 μM	
		7-(3,4-dihydroxy-5-methoxyphenyl)-5-hydroxy-1-(4-hydroxy-3-methoxyphenyl) heptan-3-one	IC_50_: 75.4 ± 6.6 μM	
		1,5-epoxy-3-hydroxy-1-(3,4-hydroxy-5-methoxyphenyl)-7-(4-hydroxy-3-methoxyphenyl) heptane	IC_50_: 174.2 ± 15.1 μM	
		5-[4-hydroxy-6-(4-hydroxyphenethyl) tetrahydro-2H-pyran-2-yl]-3-methoxybenzene-1,2-diol	IC_50_: 247.9 ± 20.1 μM	
		Curcumin	IC_50_: 33.5 ± 10.1 μM	
	Cytotoxic effect on Hep-2 (human laryngeal carcinoma cell lines)	Zerumbone	IC50: 15 μM	[39]
	Anti-proliferative effect on HepG2 (human liver cancer cell lines)	Zerumbone	IC_50_: 3.45 ± 0.026 μg/ml	[40]
	Cytotoxic effect on 16 human oral squamous cell carcinoma lines	Zerumbone	IC_50_: average 2 µM; range: 0.8–4.9 µM	
	Cytotoxic effect on DU145 (human prostate cancer cell lines)	Zerumbone	IC_50_: 24 μM	[41]
	Cytotoxic effect on HCT116 (human colorectal cancer cell lines)	Zerumbone	IC_50_: 30 ± 1.5 μM	[42]
	Cytotoxic effect on SW620 (human colorectal cancer cell lines)	Zerumbone	IC_50_: > 46 μM	
	Cytotoxic effect on MCF-7 (human breast cancer cell lines)	Zerumbone	IC_50_: 23.0 μg/ml	[43]
	Cytotoxic effect on MDA-MB 231 (human breast cancer cell lines)	Zerumbone	IC_50_: 24.3 μg/ml	
	Cytotoxic effect on Hela (human cervical cancer cell lines)	Zerumbone	IC_50_: 6.4 μg/mL	
		6-gingerol	IC_50_: 126.89 μM	[44]
		6-dehydrogingerdione	IC_50_: 62.5 ± 4.7 μM	[45]
		Zingerone	IC_50_: 114.6 ± 9.3 μM	
		10-gingerol	IC_50_: 52.4 ± 7.1 μM	
		3,5-dioxo-1,7-bis(3-methoxy-4-hydroxy)-phenyl-heptane	IC_50_: 86.8 ± 10.5 μM	
		Gingerenone A	IC_50_: 15.4 ± 3.2 μM	
		3,5-diacetoxy-1-(3-methoxy-4,5-dihydroxy-phenyl)-7-(4-hydroxy-3-methoxyphenyl) heptane	IC_50_: 110.0 ± 9.8 μM	
		1,7-bis(4-hydroxy-3-methoxyphenyl) heptane-3,5-diol	IC_50_: 191.0 ± 16.5 μM	
		7-(3,4-dihydroxy-5-methoxyphenyl)-5-hydroxy-1-(4-hydroxy-3-methoxyphenyl) heptan-3-one	IC_50_: 133.2 ± 16.1 μM	
		5-[4-hydroxy-6-(4-hydroxyphenethyl) tetrahydro-2H-pyran-2-yl]-3-methoxybenzene-1,2-diol	IC_50_: 231.8 ± 13.7 μM	
		Curcumin	IC_50_: 18.9 ± 2.8 μM	
	Inhibitory effect on epstein-barr virus (human herpesvirus 4)	Zerumbone	IC_50_: 0.14 μM	[46]
	Cytotoxic effect on KB (human oral epithelial cancer cell lines)	6-shogaol	IC_50_: 7.4 ± 2.2 μM	[37]
		6-dehydrogingerdione	IC_50_: 229.5 ± 17.5 μM	
		10-gingerol	IC_50_: 89.5 ± 8.7 μM	
		3,5-dioxo-1,7-bis(3-methoxy-4-hydroxy)-phenyl-heptane	IC_50_: 27.7 ± 2.7 μM	
		Gingerenone A	IC_50_: 8.8 ± 2.6 μM	
		Curcumin	IC_50_: 34.7 ± 6.7 μM	
		3,5-diacetoxy-1-(3-methoxy-4,5-dihydroxy-phenyl)-7-(4-hydroxy-3-methoxyphenyl) heptane	IC_50_: 75.1 ± 10.5 μM	
		7-(3,4-dihydroxy-5-methoxyphenyl)-5-hydroxy-1-(4-hydroxy-3-methoxyphenyl) heptan-3-one	IC_50_: 90.3 ± 10.1 μM	
		1,5-epoxy-3-hydroxy-1-(3,4-hydroxy-5-methoxyphenyl)-7-(4-hydroxy-3-methoxyphenyl) heptane	IC_5_: 78.5 ± 11.3 μM	
		Curcumin	IC_50_: 34.7 ± 6.7 μM	
	Cytotoxic effect on HT29 (human colorectal cancer cell lines)	Zerumbone	IC_50_: 38.8 ± 1.2 μM	[45]

IC_50_: the half maximal inhibitory concentration.

## Data Availability

Not applicable.

## Supplementary Materials

The following supporting information can be downloaded: https://www.mdpi.com/article/10.3390/molecules27092826/s1, Table S1: A comprehensive list of chemical constituents of *Zingiber* plants.; Table S2: Antimicrobial effect of *Zingiber* plants.; Table S3: Larvicidal effect of *Zingiber* plants. References from [77,78,79,80,81,82,83,84,85,86,87,88,89,90,91,92,93,94,95,96,97,98,99,100,101,102,103,104,105,106,107,108,109,110,111,112,113,114,115,116,117,118,119,120,121,122,123,124,125,126,127,128] are cited in the Supplementary Materials.

## Sample Availability

Samples of the compounds are not available from the authors.
