# Supplementary material for: Plants of the Genus Zingiber: A Review of Their Ethnomedicine, Phytochemistry and Pharmacology
Source: Molecules. 2022 Apr 29;27(9):2826. doi: 10.3390/molecules27092826 (PMC9103766; doi:10.3390/molecules27092826)
Supplement: Supplementary file 1 [file molecules-27-02826-s001.zip › molecules-1664822-supplementary.pdf]

molecules

MDPI

*Supplementary Materials*

# Plants of the Genus Zingiber: A Review of Their Ethnomedicine, Phytochemistry and Pharmacology

**Miao Deng [1], Xuan Yun [2], Shurui Ren [1], Zhixing Qing [2,*] and Fenglian Luo [1,*]**

[1] College of Food Science and Technology, Hunan Agricultural University, Changsha 410128, China; dengmiao1999@163.com (M.D.); zsb@hunau.net (S.R.)
[2] Hunan Co-Innovation Center for Utilization of Botanical Functional Ingredients, College of Veterinary Medicine, Hunan Agricultural University, Changsha 410128, China; zengjianguo@hunau.edu.cn
* Correspondence: qingzhixing@hunau.edu.cn (Z.Q.); luofenglian@hunau.edu.cn (F.L.); Tel: +86-134-6771-5114 (Z.Q. & F.L.); Fax: +86-0731-8461-7093 (Z.Q. & F.L.)

**Table S1.** A comprehensive list of chemical constituents of *Zingiber* plants.

| No. | Compounds | Formula | MW | Sources | References |
|---|---|---|---|---|---|
| | *Volatile oils* | | | | |
| 1 | Zerumbone | $C_{16}H_{24}O$ | 232.18 | *Z. zerumbet* | [77] |
| 2 | Eugenyl acetate | $C_{12}H_{14}O_3$ | 206.09 | *Z. zerumbet* | [85] |
| 3 | β-elemenone | $C_{15}H_{22}O$ | 218.17 | *Z. zerumbet* | [85] |
| 4 | 2-nonanone | $C_9H_{18}O$ | 142.14 | *Z. zerumbet* | [87] |
| | | | | *Z. officinale* | [88] |
| 5 | 2-heptanol acetate | $C_{11}H_{22}O_2$ | 186.16 | *Z. zerumbet* | [87] |
| | | | | *Z. officinale* | [89] |
| 6 | 1,5-heptadiene | $C_7H_{12}$ | 96.09 | *Z. zerumbet* | [87] |
| 7 | Lavandulyl acetate | $C_{10}H_{16}O_2$ | 168.12 | *Z. zerumbet* | [87] |
| 8 | Eugenol | $C_{10}H_{12}O_2$ | 164.08 | *Z. zerumbet* | [87] |
| 9 | trans-isoeugenol | $C_{10}H_{12}O_2$ | 164.08 | *Z. officinale* | [90] |
| 10 | Cyclohexanemethanol | $C_7H_{14}O$ | 114.10 | *Z. zerumbet* | [87] |
| 11 | Methyl salicylate | $C_8H_8O_3$ | 152.05 | *Z. zerumbet* | [91] |
| 12 | β-sinensal | $C_{15}H_{22}O$ | 218.17 | *Z. zerumbet* | [85] |
| 13 | Cinerone | $C_{10}H_{14}O$ | 150.10 | *Z. zerumbet* | [92] |
| 14 | Phytone | $C_{17}H_{34}O$ | 254.26 | *Z. zerumbet* | [92] |
| 15 | Methy leugenol | $C_{11}H_{14}O_2$ | 178.10 | *Z. corallinum Hance* | [83] |
| | | | | *Z. officinale* | [81] |
| 16 | c-3,4 -dimethoxy cinnamyl aldehyde | $C_{11}H_{12}O_3$ | 192.08 | *Z. corallinum Hance* | [83] |
| 17 | Methyl iso-eugenol | $C_{11}H_{14}O_2$ | 178.10 | *Z. corallinum Hance* | [83] |
| 18 | t-3,5-dimethoxy alkyne cinnaldehyde | $C_{11}H_{10}O_3$ | 190.06 | *Z. corallinum Hance* | [83] |
| 19 | Apiol | $C_{12}H_{14}O_4$ | 222.09 | *Z. corallinum Hance* | [83] |
| 20 | 3-methoxy -4-hydroxy methyl cinnamate | $C_{10}H_{10}O_4$ | 194.10 | *Z. corallinum Hance* | [83] |

| | | | | | |
|---|---|---|---|---|---|
| 21 | 3,4-dimethoxybenzaldehyde | $C_9H_{10}O_3$ | 166.06 | *Z. corallinum Hance* | [93] |
| 22 | Methyl ferulate | $C_{11}H_{12}O_4$ | 208.07 | *Z. corallinum Hance* | [94] |
| 23 | Sabinen | $C_{10}H_{18}$ | 138.14 | *Z. corallinum Hance* | [94] |
| 24 | Myristicin | $C_{11}H_{12}O_3$ | 192.08 | *Z. corallinum Hance* | [94] |
| 25 | 1,4-bis(methoxy)-triquinacene | $C_{12}H_{14}O_2$ | 190.10 | *Z. corallinum Hance* | [95] |
| 26 | 1,4,7-tris(methoxy)-triquinacene | $C_{13}H_{16}O_3$ | 220.11 | *Z. corallinum Hance* | [94] |
| 27 | Linalyl acetate | $C_{13}H_{22}O_2$ | 210.16 | *Z. corallinum Hance* | [78] |
| 28 | 2-allyl-1,4-dimethoxy-3-methyl-benzene | $C_{12}H_{16}O_2$ | 192.12 | *Z. corallinum Hance* | [78] |
| 29 | 5-(4-methoxyphenyl)-2,4-pentadienoic acid | $C_{12}H_{12}O_3$ | 204.08 | *Z. corallinum Hance* | [78] |
| 30 | 1-acetyl-2-(2,3,4-trimethoxyphenyl)-ethylene | $C_{13}H_{16}O_4$ | 236.10 | *Z. corallinum Hance* | [78] |
| 31 | 5,7,8-trimethyl-6-coumarinyl ester-acetic acid | $C_{14}H_{14}O_4$ | 246.09 | *Z. corallinum Hance* | [78] |
| 32 | 2-naphthalenecarboxylic acid | $C_{14}H_{18}O_4$ | 250.12 | *Z. corallinum Hance* | [78] |
| 33 | Palmitic acid | $C_{16}H_{32}O_2$ | 256.24 | *Z. corallinum Hance* | [96] |
| | | | | *Z. striolatum Diels* | [23] |
| | | | | *Z. officinale* | [23] |
| 34 | 2,2,3-trimethyl-3-cyclopentene-1-acetaldehyde | $C_{10}H_{16}O$ | 152.12 | *Z. striolatum Diels* | [84] |
| 35 | 4-isopropyl-2-cyclohexenyl-1-one | $C_{11}H_{18}O$ | 166.14 | *Z. striolatum Diels* | [84] |
| 36 | Piperonyl alcohol | $C_8H_8O_3$ | 152.05 | *Z. striolatum Diels* | [84] |
| 37 | p-isopropylphenol | $C_9H_{12}O$ | 136.09 | *Z. striolatum Diels* | [84] |

| | | | | | |
|---|---|---|---|---|---|
| 38 | 4-(1,3-Benzodioxol-5-yl) -2-butanone | $C_{10}H_{10}O_3$ | 178.06 | *Z. striolatum Diels* | [84] |
| 39 | 1,3-p-Menthadien-7-al | $C_{10}H_{14}O$ | 150.10 | *Z. striolatum Diels* | [84] |
| 40 | Pentadecane | $C_{15}H_{32}$ | 212.25 | *Z. striolatum Diels* | [84] |
| 41 | Tributyl phosphate | $C_{12}H_{27}O_4P$ | 266.16 | *Z. striolatum Diels* | [84] |
| 42 | Hexahydroaplotaxene 1-heptadecene | $C_{18}H_{36}$ | 252.28 | *Z. striolatum Diels* | [84] |
| 43 | Heptadecane | $C_{18}H_{38}$ | 254.30 | *Z. striolatum Diels* | [84] |
| 44 | 4-isopropylbenzyl alcohol | $C_{10}H_{14}O$ | 150.10 | *Z. striolatum Diels* | [84] |
| 45 | Methyl 14-methylpentadecanoate | $C_{17}H_{34}O_2$ | 270.26 | *Z. striolatum Diels* | [23] |
| 46 | Methyl linolenate | $C_{19}H_{32}O_2$ | 292.24 | *Z. striolatum Diels* | [23] |
| 47 | (Z)-docos-13-enamide | $C_{22}H_{43}N0$ | 337.33 | *Z. striolatum Diels* | [23] |
| 48 | 2-heptanol | $C_7H_{16}O$ | 116.12 | *Z. officinale* | [81] |
| 49 | 6-methyl-5-hep-ten-2-one | $C_8H_{14}O$ | 126.10 | *Z. officinale* | [81] |
| 50 | Octanal | $C_8H_{16}O$ | 128.12 | *Z. officinale* | [81] |
| 51 | Rosefuran epoxide | $C_{10}H_{14}O_2$ | 166.10 | *Z. officinale* | [81] |
| 52 | Decanal | $C_{10}H_{20}O$ | 156.15 | *Z. officinale* | [86] |
| 53 | 2-Octenal | $C_8H_{14}O$ | 126.10 | *Z. officinale* | [24] |
| 54 | 1-cyclopropylpentane | $C_8H_{16}$ | 112.13 | *Z. officinale* | [24] |
| 55 | 2-undecanone | $C_{11}H_{22}O$ | 170.17 | *Z. officinale* | [24] |
| 56 | 4-(1-methoxyethyl)phenol | $C_9H_{12}O_2$ | 152.08 | *Z. officinale* | [99] |
| 57 | Furfural | $C_5H_4O_2$ | 96.02 | *Z. officinale* | [88] |
| 58 | Benzaldehyde | $C_7H_6O$ | 106.04 | *Z. officinale* | [88] |
| 59 | Phenylacetaldehyde | $C_8H_8O$ | 120.06 | *Z. officinale* | [88] |
| 60 | Cuminaldehyde | $C_{10}H_{12}O$ | 148.09 | *Z. officinale* | [88] |
| 61 | 3-butylfuranbutanal | $C_7H_{14}O$ | 114.10 | *Z. officinale* | [102] |
| 62 | Methyl valeraldehyde | $C_6H_{12}O$ | 100.09 | *Z. officinale* | [102] |
| 63 | Alpha,P-dimethylstyrene | $C_{10}H_{12}$ | 132.09 | *Z. officinale* | [102] |
| 64 | 3-(4-methylpent-3-enyl)furan | $C_{10}H_{14}O$ | 150.10 | *Z. officinale* | [102] |
| | | | | *Z. mioga* | [82] |
| 65 | Dibutyl phthalate | $C_{16}H_{22}O_4$ | 278.15 | *Z. officinale* | [102] |
| 66 | Diisobutyl phthalate | $C_{16}H_{22}O_4$ | 278.15 | *Z. officinale* | [103] |
| 67 | oct-1-ene | $C_8H_{16}$ | 112.13 | *Z. officinale* | [104] |
| 68 | 2-hexenal, 2-ethyl- | $C_8H_{14}O$ | 126.10 | *Z. officinale* | [104] |
| 69 | 3,5-heptadiene-2-ol | $C_7H_{12}O$ | 112.09 | *Z. officinale* | [104] |
| 70 | 5-methyl-2(5H)-furanone | $C_9H_{14}O_2$ | 98.04 | *Z. officinale* | [104] |
| 71 | Dodecanal | $C_{12}H_{24}O$ | 184.18 | *Z. officinale* | [104] |
| 72 | Pentadecanal | $C_{15}H_{30}O$ | 226.23 | *Z. officinale* | [104] |
| | | | | *Z. mioga* | [82] |

| | | | | | |
|---|---|---|---|---|---|
| 73 | 2,3,5,6-tetramethylphenol | $C_{10}H_{14}O$ | 150.10 | *Z. officinale* | [106] |
| 74 | Vinylcyclohexane | $C_8H_{14}$ | 110.11 | *Z. officinale* | [106] |
| 75 | Isomethyleugenol | $C_{11}H_{14}O_2$ | 178.10 | *Z. officinale* | [106] |
| 76 | 2-amino-7-methyl-1H-pteridin-4-one | $C_7H_7N_5O$ | 177.07 | *Z. officinale* | [106] |
| 77 | Carvacrol | $C_{10}H_{14}O$ | 150.10 | *Z. striolatum Diels* | [84] |
| | | | | *Z. mioga* | [82] |
| 78 | Dodecanoic acid | $C_{12}H_{24}O_2$ | 200.18 | *Z. officinale* | [106] |
| 79 | 1-(2-hydroxy-4,6-dimethoxy)acetophenone | $C_{10}H_{12}O_4$ | 196.07 | *Z. officinale* | [106] |
| 80 | 1-benzylindole | $C_{15}H_{13}N$ | 207.10 | *Z. officinale* | [106] |
| 81 | Benzoic acid | $C_7H_6O_2$ | 122.04 | *Z. officinale* | [60] |
| 82 | 2-decenol | $C_{10}H_{20}O$ | 156.15 | *Z. officinale* | [107] |
| 83 | 3-hydroxy-4-methoxymandelic Acid | $C_9H_{10}O_5$ | 198.05 | *Z. officinale* | [107] |
| 84 | Ethyl (Z)-3-(4-methoxyphenyl)prop-2-enoate | $C_{12}H_{14}O_3$ | 206.09 | *Z. officinale* | [108] |
| 85 | 4-(1-Methylethyl)-2-cyclohexen-1-one | $C_9H_{14}O$ | 138.10 | *Z. officinale* | [109] |
| 86 | Sec-butanol | $C_4H_{10}O$ | 74.07 | *Z. officinale* | [110] |
| 87 | 1,1-dimethyl-2-butene | $C_6H_{12}$ | 84.09 | *Z. officinale* | [110] |
| 88 | 2-ethoxybutane | $C_6H_{14}O$ | 102.10 | *Z. officinale* | [110] |
| 89 | 2-isopropoxyethanol | $C_5H_{12}O_2$ | 104.08 | *Z. officinale* | [110] |
| 90 | 2,4,5-trimethyl-1,3-dioxolane | $C_6H_{12}O2$ | 116.08 | *Z. officinale* | [110] |
| 91 | 2-ethoxypentane | $C_7H_{16}O$ | 116.12 | *Z. officinale* | [110] |
| 92 | 1-propoxy-2-propanol | $C_6H_{14}O_2$ | 118.10 | *Z. officinale* | [110] |
| 93 | 2-methyl-3-phenylpropan-1-ol | $C_{10}H_{14}O$ | 150.10 | *Z. officinale* | [80] |
| 94 | 1-phenylpropane-1,3-diol | $C_9H_{12}O_2$ | 152.08 | *Z. officinale* | [80] |
| 95 | 2-pentylcyclopent-2-en-1-one | $C_{10}H_{16}O$ | 152.12 | *Z. officinale* | [80] |
| 96 | 2-tridecanol | $C_{13}H_{28}O$ | 200.21 | *Z. officinale* | [80] |
| 97 | 2-Isopropenyl-5-methylhex-4-enylacetat | $C_{12}H_{20}O_2$ | 196.15 | *Z. officinale* | [80] |
| 98 | Ethyl formate | $C_3H_6O_2$ | 74.04 | *Z. mioga* | [82] |
| 99 | o-cymene | $C_{10}H_{14}$ | 134.11 | *Z. zerumbet* | [77] |
| | | | | *Z. corallinum Hance* | [78] |
| | | | | *Z. officinale* | [79] |
| 100 | m-cymene | $C_{10}H_{14}$ | 134.11 | *Z. corallinum Hance* | [78] |
| 101 | p-cymene | $C_{10}H_{14}$ | 134.11 | *Z. zerumbet* | [87] |
| | | | | *Z. corallinum Hance* | [83] |
| | | | | *Z. striolatum Diels* | [84] |
| | | | | *Z. officinale* | [81] |
| | | | | *Z. mioga* | [82] |
| 102 | p-cymen-8-ol | $C_{10}H_{14}O$ | 150.10 | *Z. zerumbet* | [91] |
| | | | | *Z. officinale* | [79] |
| | | | | *Z. mioga* | [82] |
| 103 | p-ethylcumene | $C_{11}H_{16}$ | 148.13 | *Z. mioga* | [82] |
| 104 | 4-isopropylcyclohexanone | $C_9H_{16}O$ | 140.12 | *Z. mioga* | [82] |
| 105 | 3-caren-10-al | $C_{10}H_{14}O$ | 150.10 | *Z. mioga* | [82] |
| 106 | (E)-3-caren-2-ol | $C_{10}H_{16}O$ | 152.12 | *Z. mioga* | [82] |
| | | ***Monoterpene*** | | | |
| 107 | Myrtenyl acetate | $C_{12}H_{18}O_2$ | 194.13 | *Z. zerumbet* | [7] |

| | | | | | |
|---|---|---|---|---|---|
| | | | | *Z. officinale* | [79] |
| | | | | *Z. mioga* | [82] |
| 108 | 3,6,6-Trimethyl-bicyclo[3.1.1]hept-2-ene | $C_{10}H_{16}$ | 136.13 | *Z. zerumbet* | [92] |
| 109 | Myrtenal | $C_{10}H_{14}O$ | 150.10 | *Z. zerumbet* | [92] |
| | | | | *Z. officinale* | [79] |
| | | | | *Z. mioga* | [82] |
| 110 | Myrtenol | $C_{10}H_{16}O$ | 152.12 | *Z. striolatum Diels* | [84] |
| | | | | *Z. officinale* | [81] |
| | | | | *Z. mioga* | [82] |
| 111 | (S)-cis-verbenol | $C_{10}H_{16}O$ | 152.12 | *Z. officinale* | [80] |
| 112 | $\alpha$-pinene | $C_{10}H_{16}$ | 136.13 | *Z. zerumbet* | [77] |
| | | | | *Z. corallinum Hance* | [78] |
| | | | | *Z. striolatum Diels* | [84] |
| | | | | *Z. officinale* | [81] |
| | | | | *Z. mioga* | [82] |
| 113 | β-elemene | $C_{15}H_{24}$ | 204.19 | *Z. zerumbet* | [7] |
| | | | | *Z. corallinum Hance* | [78] |
| | | | | *Z. striolatum Diels* | [84] |
| | | | | *Z. officinale* | [81] |
| | | | | *Z. mioga* | [82] |
| 114 | Carvopinone | $C_{10}H_{14}O$ | 150.10 | *Z. striolatum Diels* | [84] |
| 115 | z-pinocarveol | $C_{10}H_{16}O$ | 152.12 | *Z. striolatum Diels* | [84] |
| | | | | *Z. mioga* | [82] |
| 116 | [(1S,4S,5S)-6,6-dimethyl-4-bicyclo[3.1.1]heptanyl]methanol | $C_{10}H_{18}O$ | 154.14 | *Z. officinale* | [102] |
| 117 | δ-3-carene | $C_{10}H_{16}$ | 136.13 | *Z. zerumbet* | [77] |
| | | | | *Z. officinale* | [79] |
| | | | | *Z. mioga* | [82] |
| 118 | Pinene oxide | $C_{10}H_{16}O$ | 152.12 | *Z. corallinum Hance* | [83] |
| 119 | $\alpha$-cyclocitral | $C_{10}H_{16}O$ | 152.12 | *Z. officinale* | [80] |
| 120 | $\alpha$-terpinolene | $C_{10}H_{18}$ | 138.14 | *Z. zerumbet* | [87] |
| | | | | *Z. corallinum Hance* | [96] |
| | | | | *Z. officinale* | [81] |
| | | | | *Z. mioga* | [82] |
| 121 | β-terpinene | $C_{10}H_{16}$ | 136.13 | *Z. corallinum Hance* | [94] |
| | | | | *Z. officinale* | [79] |
| | | | | *Z. mioga* | [82] |

| | | | | | |
|---|---|---|---|---|---|
| 122 | 2-cyclohexen-1-ol, 1-methyl-4-(1-methylethyl)-, trans- | $C_{10}H_{18}O$ | 154.14 | *Z. corallinum Hance* | [78] |
| 123 | trans-carveol | $C_{10}H_{16}O$ | 152.12 | *Z. corallinum Hance* | [78] |
| | | | | *Z. striolatum Diels* | [84] |
| | | | | *Z. mioga* | [82] |
| 124 | Carvone | $C_{10}H_{14}O$ | 150.10 | *Z. striolatum Diels* | [84] |
| | | | | *Z. mioga* | [82] |
| 125 | Perillaldehyde | $C_{10}H_{14}O$ | 150.10 | *Z. striolatum Diels* | [84] |
| 126 | Limonene | $C_{10}H_{16}$ | 136.13 | *Z. zerumbet* | [77] |
| | | | | *Z. corallinum Hance* | [83] |
| | | | | *Z. striolatum Diels* | [84] |
| | | | | *Z. officinale* | [81] |
| | | | | *Z. mioga* | [82] |
| 127 | Perillylalcohol | $C_{10}H_{16}O$ | 152.12 | *Z. striolatum Diels* | [84] |
| 128 | Terpinen-4-ol | $C_{10}H_{18}O$ | 154.14 | *Z. zerumbet* | [77] |
| | | | | *Z. corallinum Hance* | [83] |
| | | | | *Z. striolatum Diels* | [84] |
| | | | | *Z. officinale* | [81] |
| 129 | c-piperitol | $C_{10}H_{18}O$ | 154.14 | *Z. corallinum Hance* | [83] |
| 130 | Cuminyl acetate | $C_{12}H_{20}O_2$ | 196.15 | *Z. mioga* | [82] |
| 131 | β -phellandrene | $C_{10}H_{16}$ | 136.13 | *Z. corallinum Hance* | [83] |
| | | | | *Z. striolatum Diels* | [84] |
| | | | | *Z. officinale* | [81] |
| | | | | *Z. mioga* | [82] |
| 132 | Dihydrocarveol | $C_{10}H_{18}O$ | 154.14 | *Z. corallinum Hance* | [78] |
| 133 | Isopulego | $C_{10}H_{18}O$ | 154.14 | *Z. officinale* | [106] |
| 134 | DL-menthol | $C_{10}H_{20}O$ | 156.15 | *Z. corallinum Hance* | [93] |
| 135 | Menthone | $C_{10}H_{18}O$ | 154.14 | *Z. officinale* | [81] |

| | | | | | |
|---|---|---|---|---|---|
| 136 | Sylvestrene | $C_{10}H_{16}$ | 136.13 | *Z. zerumbet* | [92] |
| 137 | Terpinyl acetate | $C_{12}H_{20}O_2$ | 196.15 | *Z. corallinum Hance* | [93] |
| 138 | Bornyl acetate | $C_{12}H_{20}O_2$ | 196.15 | *Z. zerumbet* | [77] |
| | | | | *Z. officinale* | [81] |
| | | | | *Z. mioga* | [82] |
| 139 | Piperitenone | $C_{10}H_{14}O$ | 150.10 | *Z. mioga* | [82] |
| 140 | 1, 4-cineol | $C_{10}H_{16}$ | 136.13 | *Z. corallinum Hance* | [83] |
| | | | | *Z. officinale* | [80] |
| 141 | cis-β-terpineol | $C_{10}H_{18}O$ | 154.14 | *Z. corallinum Hance* | [95] |
| 142 | α-terpinene | $C_{10}H_{16}$ | 136.13 | *Z. corallinum Hance* | [83] |
| | | | | *Z. striolatum Diels* | [84] |
| | | | | *Z. mioga* | [82] |
| 143 | p-menth-2,8-dienol | $C_{10}H_{16}O$ | 152.12 | *Z. striolatum Diels* | [84] |
| 144 | Phellandral | $C_{10}H_{16}O$ | 152.12 | *Z. mioga* | [82] |
| 145 | 2-cyclohexen-1-ol, 1-methyl-4-(1-methylethyl)-, cis- | $C_{10}H_{18}O$ | 154.14 | *Z. corallinum Hance* | [78] |
| | | | | *Z. striolatum Diels* | [84] |
| | | | | *Z. mioga* | [82] |
| 146 | α-phellandrene | $C_{10}H_{16}$ | 136.13 | *Z. zerumbet* | [7] |
| | | | | *Z. corallinum Hance* | [83] |
| | | | | *Z. striolatum Diels* | [84] |
| | | | | *Z. officinale* | [81] |
| | | | | *Z. mioga* | [82] |
| 147 | γ-terpinene | $C_{10}H_{16}$ | 136.13 | *Z. zerumbet* | [7] |
| | | | | *Z. corallinum Hance* | [78] |
| | | | | *Z. striolatum Diels* | [84] |
| | | | | *Z. officinale* | [102] |
| | | | | *Z. mioga* | [82] |
| 148 | Fenchone | $C_{10}H_{16}O$ | 152.12 | *Z. zerumbet* | [77] |
| | | | | *Z. mioga* | [82] |
| 149 | Camphene | $C_{10}H_{16}$ | 136.13 | *Z. zerumbet* | [77] |
| | | | | *Z. corallinum Hance* | [95] |

| | | | | | |
|---|---|---|---|---|---|
| | | | | *Z. striolatum Diels* | [84] |
| | | | | *Z. officinale* | [81] |
| | | | | *Z. mioga* | [82] |
| 150 | Tricyclene | $C_{10}H_{18}$ | 138.14 | *Z. zerumbet* | [7] |
| | | | | *Z. officinale* | [81] |
| 151 | β-fenchene | $C_{10}H_{16}$ | 136.13 | *Z. corallinum Hance* | [94] |
| | | | | *Z. mioga* | [82] |
| 152 | Linalool | $C_{10}H_{18}O$ | 154.14 | *Z. zerumbet* | [77] |
| | | | | *Z. corallinum Hance* | [83] |
| | | | | *Z. striolatum Diels* | [84] |
| | | | | *Z. officinale* | [80] |
| | | | | *Z. mioga* | [82] |
| 153 | Borneol | $C_{10}H_{18}O$ | 154.14 | *Z. zerumbet* | [77] |
| | | | | *Z. striolatum Diels* | [84] |
| | | | | *Z. officinale* | [81] |
| | | | | *Z. mioga* | [82] |
| 154 | α-terpineol | $C_{10}H_{18}O$ | 154.14 | *Z. zerumbet* | [77] |
| | | | | *Z. corallinum Hance* | [78] |
| | | | | *Z. officinale* | [81] |
| | | | | *Z. mioga* | [82] |
| 155 | α-fenchene | $C_{10}H_{16}$ | 136.13 | *Z. mioga* | [82] |
| 156 | 1,8-cineole | $C_{10}H_{18}O$ | 154.14 | *Z. zerumbet* | [77] |
| | | | | *Z. officinale* | [81] |
| 157 | 2-carene | $C_{10}H_{16}$ | 136.13 | *Z. officinale* | [79] |
| 158 | Carene-4 | $C_{10}H_{16}$ | 136.13 | *Z. corallinum Hance* | [83] |
| | | | | *Z. officinale* | [79] |
| 159 | Ascaridole epoxide | $C_{10}H_{16}O_2$ | 168.12 | *Z. corallinum Hance* | [78] |
| 160 | Caran-2-ol | $C_{10}H_{18}O$ | 154.14 | *Z. corallinum Hance* | [94] |
| 161 | Limonene 1,2-epoxide | $C_{10}H_{16}O$ | 152.12 | *Z. officinale* | [80] |
| 162 | Neral | $C_{10}H_{16}O$ | 152.12 | *Z. zerumbet* | [81] |
| | | | | *Z. officinale* | [81] |
| 163 | Nerol | $C_{10}H_{18}O$ | 154.14 | *Z. officinale* | [24] |
| 164 | cis-2,6-Dimethyl-2,6-octadiene | $C_{10}H_{18}$ | 138.14 | *Z. officinale* | [24] |
| 165 | Geranic acid | $C_{10}H_{16}O_2$ | 168.12 | *Z. officinale* | [24] |
| 166 | Neryl propionate | $C_{13}H_{22}O_2$ | 210.16 | *Z. officinale* | [24] |
| 167 | Neryl acetate | $C_{12}H_{20}O_2$ | 196.15 | *Z. officinale* | [24] |
| 168 | Citronellal | $C_{10}H_{18}O$ | 154.14 | *Z. zerumbet* | [87] |
| | | | | *Z. officinale* | [81] |
| 169 | Citronellyl acetate | $C_{12}H_{22}O_2$ | 198.16 | *Z. zerumbet* | [87] |

| | | | | | |
|---|---|---|---|---|---|
| | | | | *Z. officinale* | [88] |
| | | | | *Z. officinale* | [81] |
| 170 | Citronellol | $C_{10}H_{20}O$ | 156.15 | *Z. officinale* | [81] |
| 171 | β-myrcene | $C_{10}H_{16}$ | 136.13 | *Z. zerumbet* | [7] |
| | | | | *Z. corallinum Hance* | [83] |
| | | | | *Z. striolatum Diels* | [84] |
| | | | | *Z. officinale* | [81] |
| | | | | *Z. mioga* | [82] |
| 172 | Ocimene | $C_{10}H_{16}$ | 136.13 | *Z. zerumbet* | [92] |
| | | | | *Z. striolatum Diels* | [84] |
| | | | | *Z. officinale* | [104] |
| 173 | Hotrienol | $C_{10}H_{16}O$ | 152.12 | *Z. officinale* | [86] |
| 174 | Linalool | $C_{10}H_{18}O$ | 154.14 | *Z. zerumbet* | [77] |
| | | | | *Z. corallinum Hance* | [83] |
| | | | | *Z. striolatum Diels* | [84] |
| | | | | *Z. officinale* | [80] |
| | | | | *Z. mioga* | [82] |
| 175 | Eucarvone | $C_{10}H_{14}O$ | 150.10 | *Z. mioga* | [82] |
| 176 | Sabinene hydrate | $C_{10}H_{18}O$ | 154.14 | *Z. corallinum Hance* | [83] |
| | | | | *Z. striolatum Diels* | [84] |
| | | | | *Z. mioga* | [82] |
| 177 | α-thujene | $C_{10}H_{14}O$ | 136.13 | *Z. corallinum Hance* | [83] |
| | | | | *Z. striolatum Diels* | [84] |
| | | | | *Z. mioga* | [82] |
| 178 | β-thujene | $C_{10}H_{16}$ | 136.13 | *Z. officinale* | [79] |
| 179 | Sabinene | $C_{10}H_{16}$ | 136.13 | *Z. zerumbet* | [7] |
| | | | | *Z. corallinum Hance* | [78] |
| | | | | *Z. striolatum Diels* | [84] |
| | | | | *Z. officinale* | [81] |
| 180 | Camphene hydrate | $C_{10}H_{18}O$ | 154.14 | *Z. zerumbet* | [77] |
| 181 | Camphene oxide | $C_{10}H_{16}O$ | 152.12 | *Z. corallinum Hance* | [83] |
| 182 | β-pinene | $C_{10}H_{16}$ | 136.13 | *Z. zerumbet* | [77] |

| | | | | | |
|---|---|---|---|---|---|
| | | | | *Z. corallinum Hance* | [78] |
| | | | | *Z. striolatum Diels* | [84] |
| | | | | *Z. officinale* | [79] |
| | | | | *Z. mioga* | [82] |
| | ***Sesquiterpene, Diterpene and Triterpene*** | | | | |
| 183 | γ-selinene | $C_{15}H_{24}$ | 204.19 | *Z. striolatum Diels* | [84] |
| 184 | Eudesma-3,7(11)-diene | $C_{15}H_{24}$ | 204.19 | *Z. officinale* | [24] |
| 185 | α-Cyperone | $C_{15}H_{22}O$ | 218.17 | *Z. zerumbet* | [105] |
| | | | | *Z. officinale* | [104] |
| 186 | β-fenchene | $C_{10}H_{16}$ | 136.13 | *Z. corallinum Hance* | [94] |
| | | | | *Z. mioga* | [82] |
| 187 | α-fenchene | $C_{10}H_{16}$ | 136.13 | *Z. mioga* | [82] |
| 188 | Farnesol | $C_{14}H_{24}O$ | 208.18 | *Z. corallinum Hance* | [94] |
| | | | | *Z. officinale* | [23] |
| | | | | *Z. mioga* | [82] |
| 189 | γ-eudesmol | $C_{15}H_{26}O$ | 220.20 | *Z. zerumbet* | [91] |
| 190 | β-eudesmol | $C_{15}H_{28}O$ | 224.21 | *Z. zerumbet* | [77] |
| | | | | *Z. officinale* | [79] |
| 191 | α-eudesmol | $C_{15}H_{26}O$ | 220.20 | *Z. zerumbet* | [91] |
| 192 | Isolongifolene | $C_{15}H_{24}$ | 204.19 | *Z. corallinum Hance* | [78] |
| 193 | α-muurolene | $C_{15}H_{24}$ | 204.19 | *Z. zerumbet* | [91] |
| 194 | δ-cadinene | $C_{15}H_{24}$ | 204.19 | *Z. zerumbet* | [7] |
| | | | | *Z. officinale* | [79] |
| 195 | β-cadinene | $C_{15}H_{24}$ | 204.19 | *Z. officinale* | [80] |
| 196 | γ-cadinene | $C_{15}H_{24}$ | 204.19 | *Z. zerumbet* | [91] |
| | | | | *Z. officinale* | [23] |
| | | | | *Z. mioga* | [82] |
| 197 | epi-bicyclosesquiphellandrene | $C_{15}H_{24}$ | 204.19 | *Z. zerumbet* | [87] |
| 198 | Miogadial | $C_{20}H_{30}O_3$ | 318.22 | *Z. mioga* | [25] |
| 199 | γ-bicyclohomofarnesal | $C_{17}H_{30}O$ | 250.23 | *Z. zerumbet* | [77] |
| 200 | epi-α-cadinol | $C_{15}H_{26}O$ | 220.20 | *Z. zerumbet* | [85] |
| 201 | Juniper camphor | $C_{15}H_{26}O$ | 222.20 | *Z. mioga* | [82] |
| 202 | α-cedrene | $C_{15}H_{24}$ | 204.19 | *Z. zerumbet* | [87] |
| | | | | *Z. officinale* | [79] |
| 203 | β-himachalene | $C_{15}H_{24}$ | 204.19 | *Z. mioga* | [82] |
| 204 | γ-gurjunene | $C_{15}H_{24}$ | 190.17 | *Z. corallinum Hance* | [78] |
| 205 | Alloaromadendrene | $C_{15}H_{24}$ | 204.19 | *Z. officinale* | [23] |
| 206 | Spathulenol | $C_{15}H_{24}O$ | 220.18 | *Z. zerumbet* | [85] |
| | | | | *Z. striolatum Diels* | [84] |
| | | | | *Z. officinale* | [86] |

| | | | | | |
|---|---|---|---|---|---|
| 207 | Palustrol(ledum) | $C_{15}H_{26}O$ | 222.20 | *Z. officinale* | [80] |
| 208 | Dehydro-aromadendrene | $C_{15}H_{22}$ | 202.17 | *Z. zerumbet* | [87] |
| 209 | α-gurjunene | $C_{15}H_{24}$ | 204.19 | *Z. zerumbet* | [7] |
| | | | | *Z. striolatum Diels* | [84] |
| | | | | *Z. officinale* | [79] |
| | | | | *Z. mioga* | [82] |
| 210 | Isofuranogermacrene | $C_{15}H_{20}O$ | 216.15 | *Z. officinale* | [80] |
| 211 | α-guaiene | $C_{15}H_{24}$ | 204.19 | *Z. officinale* | [81] |
| | | | | *Z. mioga* | [82] |
| 212 | Champacol | $C_{15}H_{26}O$ | 222.20 | *Z. officinale* | [86] |
| 213 | (1aR,4R,7R,7bS)-1,1,4,7-tetramethyl-1a,2,3,4,5,6,7,7b-octahydrocyclopropa[e]azulene | $C_{15}H_{24}$ | 204.19 | *Z. officinale* | [107] |
| 214 | Ledane | $C_{15}H_{26}$ | 206.20 | *Z. mioga* | [82] |
| 215 | Curcumadiol | $C_{15}H_{26}O_2$ | 238.19 | *Z. mioga* | [112] |
| 216 | β-patchoulene | $C_{15}H_{24}$ | 204.19 | *Z. officinale* | [79] |
| | | | | *Z. mioga* | [82] |
| 217 | α-patchoulene | $C_{15}H_{24}$ | 204.19 | *Z. officinale* | [81] |
| 218 | Widdrol | $C_{15}H_{26}O$ | 222.20 | *Z. mioga* | [82] |
| 219 | α-zingiberene | $C_{15}H_{24}$ | 204.19 | *Z. zerumbet* | [87] |
| | | | | *Z. officinale* | [81] |
| | | | | *Z. mioga* | [82] |
| 220 | α-copaene | $C_{15}H_{24}$ | 204.19 | *Z. zerumbet* | [7] |
| | | | | *Z. striolatum Diels* | [84] |
| | | | | *Z. officinale* | [88] |
| 221 | γ-elemene | $C_{15}H_{24}$ | 204.19 | *Z. corallinum Hance* | [93] |
| | | | | *Z. officinale* | [86] |
| 222 | g-elemene | $C_{15}H_{24}$ | 204.19 | *Z. zerumbet* | [87] |
| 223 | Cedrenol | $C_{15}H_{24}O$ | 220.18 | *Z. officinale* | [80] |
| 224 | ar-curcumene | $C_{15}H_{22}$ | 202.17 | *Z. zerumbet* | [87] |
| | | | | *Z. corallinum Hance* | [78] |
| | | | | *Z. officinale* | [81] |
| | | | | *Z. mioga* | [82] |
| 225 | β-sesquiphellandrene | $C_{15}H_{24}$ | 204.19 | *Z. corallinum Hance* | [93] |
| | | | | *Z. mioga* | [82] |
| 226 | (E, E)-α-farnesene | $C_{15}H_{24}$ | 204.19 | *Z. zerumbet* | [87] |
| | | | | *Z. corallinum Hance* | [94] |
| | | | | *Z. officinale* | [23] |
| 227 | c-β-farnesene | $C_{15}H_{24}$ | 204.19 | *Z. corallinum Hance* | [83] |
| | | | | *Z. officinale* | [23] |
| | | | | *Z. mioga* | [82] |
| 228 | Farnesol | $C_{14}H_{24}O$ | 208.18 | *Z. corallinum Hance* | [94] |

| | | | | | |
|---|---|---|---|---|---|
| | | | | *Z. officinale* | [23] |
| | | | | *Z. mioga* | [82] |
| 229 | Farnesal | $C_{15}H_{24}O$ | 220.18 | *Z. officinale* | [109] |
| 230 | Farnesyl acetate | $C_{17}H_{28}O_2$ | 264.21 | *Z. officinale* | [90] |
| 231 | Geranyl linalool | $C_{20}H_{34}O$ | 290.26 | *Z. officinale* | [86] |
| 232 | D-nerolidol | $C_{16}H_{30}O$ | 224.21 | *Z. zerumbet* | [7] |
| 233 | Nerolidol | $C_{16}H_{28}O$ | 236.21 | *Z. zerumbet* | [91] |
| | | | | *Z. officinale* | [81] |
| 234 | 2,5-dihydroxybisabola-3,10-diene | $C_{15}H_{26}O_2$ | 238.19 | *Z. officinale* | [6] |
| 235 | 4,5-dihydroxybisabola-2,10-diene | $C_{15}H_{26}O_2$ | 238.19 | *Z. officinale* | [111] |
| 236 | Sesquisabinene hydrate | $C_{15}H_{26}O$ | 222.20 | *Z. officinale* | [23] |
| 237 | Agarospirol | $C_{15}H_{26}O$ | 222.20 | *Z. mioga* | [82] |
| 238 | α-panasinsene | $C_{15}H_{24}$ | 204.19 | *Z. officinale* | [89] |
| 239 | β-panasinsene | $C_{15}H_{24}$ | 204.19 | *Z. officinale* | [89] |
| 240 | Patchouli alcohol | $C_{15}H_{26}O$ | 222.20 | *Z. officinale* | [81] |
| 241 | Neoclovene | $C_{15}H_{24}$ | 204.19 | *Z. officinale* | [24] |
| 242 | Germacrene D | $C_{15}H_{24}$ | 204.19 | *Z. zerumbet* | [87] |
| | | | | *Z. officinale* | [79] |
| 243 | Germacrene B | $C_{15}H_{24}$ | 204.19 | *Z. corallinum Hance* | [93] |
| 244 | β-cubebene | $C_{15}H_{24}$ | 204.19 | *Z. striolatum Diels* | [84] |
| 245 | (+)-cuparene | $C_{15}H_{22}$ | 202.17 | *Z. corallinum Hance* | [78] |
| 246 | α-cuparenol | $C_{15}H_{22}O$ | 218.17 | *Z. officinale* | [80] |
| 247 | α-caryophyllene | $C_{15}H_{24}$ | 204.19 | *Z. zerumbet* | [92] |
| | | | | *Z. striolatum Diels* | [84] |
| 248 | α-humulene | $C_{15}H_{24}$ | 204.19 | *Z. zerumbet* | [77] |
| 249 | β-humulene | $C_{15}H_{24}$ | 204.19 | *Z. officinale* | [108] |
| 250 | β-caryophylene | $C_{15}H_{24}$ | 204.19 | *Z. zerumbet* | [77] |
| | | | | *Z. corallinum Hance* | [93] |
| | | | | *Z. officinale* | [81] |
| 251 | Elemol | $C_{15}H_{26}O$ | 222.20 | *Z. zerumbet* | [91] |
| | | | | *Z. officinale* | [81] |
| | | | | *Z. mioga* | [82] |
| 252 | Humulene epoxide I | $C_{15}H_{24}O$ | 220.18 | *Z. zerumbet* | [77] |
| 253 | Humulene epoxide II | $C_{15}H_{24}O$ | 220.18 | *Z. zerumbet* | [77] |
| | | | | *Z. striolatum Diels* | [84] |
| 254 | Humulene epoxide III | $C_{15}H_{24}O$ | 220.18 | *Z. zerumbet* | [77] |
| 255 | Caryophyllene oxide | $C_{15}H_{24}O$ | 220.18 | *Z. zerumbet* | [77] |
| | | | | *Z. striolatum Diels* | [84] |
| | | | | *Z. officinale* | [20] |
| | | | | *Z. mioga* | [82] |
| 256 | (E)-β-bergamotene | $C_{15}H_{24}$ | 204.19 | *Z. zerumbet* | [87] |
| | | | | *Z. mioga* | [82] |

| | | | | | |
|---|---|---|---|---|---|
| 257 | 1,5,5,8-tetramethyl-12-oxabicyclo 9.1.0-dodeca-3,7-diene | $C_{15}H_{24}O$ | 220.18 | *Z. mioga* | [82] |
| 258 | $\alpha$-bisabolol | $C_{15}H_{26}O$ | 222.20 | *Z. officinale* | [86] |
| 259 | Squalene | $C_{30}H_{50}$ | 410.39 | *Z. officinale*<br>*Z. mioga* | [106]<br>[82] |
| 260 | Galanal A | $C_{20}H_{30}O_3$ | 318.22 | *Z. mioga* | [113] |
| 261 | Galanal B | $C_{20}H_{30}O_3$ | 318.22 | *Z. mioga* | [113] |
| | ***Flavonoids*** | | | | |
| 262 | Kaempferol-3,4',7-O-trimethylether | $C_{18}H_{16}O_6$ | 328.09 | *Z. zerumbet* | [114] |
| 263 | Kaempferol-3-O-methylether | $C_{16}H_{12}O_6$ | 300.06 | *Z. zerumbet* | [114] |
| 264 | Kaempferol-3,4'-O-dimethylether | $C_{17}H_{14}O_6$ | 314.08 | *Z. zerumbet* | [114] |
| 265 | Kaempferol-3-O-(4-O-acetyl-(x-L-rhamnopyranoside) | $C_{23}H_{22}O_{11}$ | 474.12 | *Z. zerumbet* | [114] |
| 266 | Kaempferol-3-O-(2,4-O-diacetyl-cz-L-rhamnopyranoside) | $C_{25}H_{24}O_{12}$ | 516.13 | *Z. zerumbet* | [114] |
| 267 | Kaempferol-3-O-(3,4-O-diacetyl--$\alpha$-L-rhamnopyranoside) | $C_{25}H_{24}O_{12}$ | 516.13 | *Z. zerumbet* | [114] |
| 268 | Quercetin-deoxyhexose- hexose | $C_{27}H_{30}O_{16}$ | 610.15 | *Z. striolatum Diels* | [54] |
| 269 | Quercetin-di-O-galloyl- hexose | $C_{35}H_{28}O_{20}$ | 768.12 | *Z. striolatum Diels* | [54] |
| 270 | Kaempferol-acetyl-deoxyhexose- hexose | $C_{29}H_{32}O_{16}$ | 636.17 | *Z. striolatum Diels* | [54] |
| 271 | Kaempferol-deoxyhexose- hexose | $C_{27}H_{30}O_{15}$ | 594.16 | *Z. striolatum Diels* | [54] |
| 272 | Dihydrokaempferol | $C_{15}H_{12}O_6$ | 288.06 | *Z. striolatum Diels* | [54] |
| 273 | Kaempferol-galloyl- hexose | $C_{28}H_{24}O_{15}$ | 600.11 | *Z. striolatum Diels* | [54] |
| 274 | Quercetin-acetyl-deoxyhexose- hexose | $C_{29}H_{32}O_{17}$ | 652.16 | *Z. striolatum Diels* | [54] |
| 275 | Methyl-quercetin-$OCH_3$ | $C_{17}H_{14}O_7$ | 330.07 | *Z. striolatum Diels* | [54] |
| 276 | Kaempferol-deoxyhexose | $C_{21}H_{20}O_{10}$ | 432.11 | *Z. striolatum Diels* | [54] |
| 277 | Methyl-quercetin-2$OCH_3$ | $C_{18}H_{16}O_7$ | 344.09 | *Z. striolatum Diels* | [54] |
| 278 | Kaempferol-pentose | $C_{20}$H18$O_{10}$ | 418.09 | *Z. striolatum Diels* | [54] |
| 279 | Quercetin-galloyl-pentose | $C_{27}H_{22}O_{15}$ | 586.10 | *Z. striolatum Diels* | [54] |
| 280 | 7,4′-dihydroxyflavanone | $C_{15}H_{10}O_4$ | 254.06 | *Z. striolatum Diels* | [54] |

| | | | | | |
|---|---|---|---|---|---|
| 281 | Quercetin-O-pentose | $C_{20}H_{18}O_{11}$ | 434.08 | *Z. striolatum Diels* | [54] |
| 282 | Quercetin-galloyl- hexose | $C_{28}H_{24}O_{16}$ | 616.11 | *Z. striolatum Diels* | [54] |
| 283 | 5, 3′-di-OH-isoflavone | $C_{15}H_{10}O_4$ | 254.06 | *Z. striolatum Diels* | [54] |
| 284 | Kaempferol-galloyl-pentose | $C_{27}H_{22}0_{14}$ | 570.10 | *Z. striolatum Diels* | [54] |
| 285 | Kaempferol-di-O-galloyl- hexose | $C_{35}H_{28}O_{19}$ | 752.12 | *Z. striolatum Diels* | [54] |
| 286 | Quercetin | $C_{15}H_{10}O_7$ | 302.04 | *Z. striolatum Diels* | [54] |
| 287 | Methyl-quercetin | $C_{16}H_{12}O_7$ | 316.06 | *Z. striolatum Diels* | [54] |
| 288 | Kaempferol | $C_{15}H_{10}O_6$ | 286.05 | *Z. striolatum Diels* | [54] |
| 289 | 5-hydro-3,7,3′,4′-tetra methoxyflavone | $C_{19}H_{18}O_7$ | 358.11 | *Z. officinale* | [115] |
| 290 | Galangin | $C_{15}H_{11}O_7$ | 303.05 | *Z. mioga* | [112] |
| 291 | Rutin | $C_{27}H_{30}O_{16}$ | 580.15 | *Z. mioga* | [112] |
| 292 | Delphinidin | $C_{15}H_{10}O_5$ | 270.05 | *Z. mioga* | [112] |
| 293 | Cyanidin | $C_{15}H_{11}O_6$ | 287.06 | *Z. mioga* | [112] |
| | | ***Diphenylheptanes*** | | | |
| 294 | Curcumin | $C_{21}H_{20}O_6$ | 368.13 | *Z. zerumbet* | [116] |
| | | | | *Z. corallinum Hance* | [94] |
| 295 | Hexahydrocurcumin | $C_{21}H_{26}O_6$ | 374.17 | *Z. officinale* | [117] |
| 296 | 3-one-5(R)-ethoxy-1-(3-methoxy-4-hydroxyphenyl)-7-(3-methoxy-4,5-dihydroxy-phenyl) heptane | $C_{23}H_{30}O_7$ | 418.20 | *Z. officinale* | [101] |
| 297 | 3-one-5(R)-ethoxy-1-(3-methoxy-4-hydroxyphenyl)-7-(3,4-dihydroxy-phenyl) heptane | $C_{22}H_{28}O_6$ | 388.19 | *Z. officinale* | [101] |
| 298 | (E)-7-(4-hydroxyphenyl)-1-heptphenyl-4-en-3-one | $C_{19}H_{20}O_2$ | 280.15 | *Z. officinale* | [115] |
| 299 | 7-(3,4-dihydroxy-phenyl)-5-hydroxy-1-phenyl-3-heptanone | $C_{19}H_{22}O_4$ | 314.15 | *Z. officinale* | [115] |
| 300 | 7-(3,4-dihydroxy-5-methoxyphenyl)-5-hydroxy-1-phenyl-3-heptanone | $C_{20}H_{24}O_5$ | 344.16 | *Z. officinale* | [115] |
| 301 | 7-(3,4-dihydroxy-5-methoxy-phenyl)-5-hydroxy-1-(4-hydroxy-3-methoxyphenyl)-3-heptanone | $C_{21}H_{26}O_7$ | 390.17 | *Z. officinale* | [115] |
| 302 | 7-(3,4-dihydroxy-5-methoxyphenyl)-5-hydroxy-1-(4-hydroxy-3-methoxyphenyl)heptan-3-one | $C_{21}H_{26}O_7$ | 390.17 | *Z. officinale* | [41] |
| 303 | 1-(4-Hydroxy-3-methoxy-phenyl)-7-(3-hydroxy-4-methoxy-phenyl)-3,5-heptanediol | $C_{21}H_{28}O_6$ | 376.19 | *Z. officinale* | [115] |
| 304 | 1,7-bis(4-hydroxy-3-methoxyphenyl)heptane-3,5-diol | $C_{21}H_{28}O_6$ | 376.19 | *Z. officinale* | [41] |
| 305 | (E)-5-hydroxy-1,7-di-phenylhept-6-en-3-one | $C_{19}H_{20}O_2$ | 280.15 | *Z. officinale* | [115] |
| 306 | (4Z,6E)-5-hydroxy-1,7-diphenylhepta-4,6-dien-3-one | $C_{19}H_{18}O_2$ | 278.13 | *Z. officinale* | [115] |
| 307 | 7-(4-hydroxy-3-methoxyphenyl)-1-(4-hydroxyphenyl)-5-methoxy-3-heptanone | $C_{21}H_{26}O_5$ | 358.18 | *Z. officinale* | [115] |

| | | | | | |
|---|---|---|---|---|---|
| 308 | 7-(4-hydroxy-3-methoxypheny)-1-(4-hydroxy-phenyl)-3-heptanone | $C_{20}H_{24}O_4$ | 328.17 | *Z. officinale* | [115] |
| 309 | 1,7-bis(4-hydroxyphenyl)-3-heptanone | $C_{19}H_{22}O_3$ | 298.16 | *Z. officinale* | [115] |
| 310 | (E)-7-(3,4-Dihydroxyphenyl)-1-(4-hydro-3-methoxypheny)-4-hepten-3-one | $C_{20}H_{22}O_5$ | 342.15 | *Z. officinale* | [115] |
| 311 | Gingerenone A | $C_{21}H_{24}O_5$ | 356.16 | *Z. officinale* | [41] |
| | | | | *Z. mioga* | [112] |
| 312 | 1-(4-hydro-3-methoxypheny)-7-phenyl-3,5-heptanedione | $C_{20}H_{22}O_4$ | 326.15 | *Z. officinale* | [115] |
| 313 | 3,5-dioxo-1,7-bis(3-methoxy-4-hydroxy)-phenyl-heptane | $C_{21}H_{24}O_6$ | 370.18 | *Z. officinale* | [41] |
| 314 | 3,5-diacetoxy-1-(3-methoxy-4,5-dihydroxy-phenyl)-7-(4-hydroxy-3-methoxyphenyl)heptane | $C_{25}H_{32}O_9$ | 476.20 | *Z. officinale* | [41] |
| 315 | 5-(6-(2-(3,4-dihydroxy-5-methoxyphenyl)-2-methoxyethyl)-4-hydroxytetrahydro-2-hpyran-2-yl)-3-methoxybenzene-1,2-diol | $C_{22}H_{28}O_9$ | 464.20 | *Z. officinale* | [111] |
| 316 | 5-(6-(3,4-dihydroxyphenethyl)-4-hydroxytetrahydro-2-hpyran-2-yl)-3-methoxybenzene-1,2-diol | $C_{20}H_{24}O_7$ | 376.15 | *Z. officinale* | [111] |
| 317 | 5-(4-hydroxy-6-(4-dihydroxyphenethyl)tetrahydro-2-hpyran-2-yl)-3-meth-oxybenzene-1,2-diol | $C_{20}H_{24}O_6$ | 360.16 | *Z. officinale* | [111] |
| 318 | 5-(4-hydroxy-6-(4-dihydroxyphenethyl)tetrahydro-2-hpyran-2-yl)-3-meth-oxy-benzene-1,2-diol | $C_{21}H_{26}O_7$ | 390.17 | *Z. officinale* | [111] |
| 319 | 1,5-epoxy-3-hydroxy-1-(3,4-hydroxy-5-methoxyphenyl)-7-(4-hydroxy-3-methoxyphenyl)heptane | $C_{21}H_{26}O_7$ | 390.17 | *Z. officinale* | [41] |
| 320 | 5-[4-hydroxy-6-(4-hydroxyphenethyl)tetrahydro-2H-pyran-2-yl]-3-methoxybenzene-1,2-dio | $C_{20}H_{24}O_6$ | 360.16 | *Z. officinale* | [41] |
| | ***Gingerol analogues*** | | | | |
| 321 | Dihydrocapsaicin | $C_{18}H_{29}NO_3$ | 307.21 | *Z. officinale* | [86] |
| 322 | Zingerone | $C_{11}H_{14}O_3$ | 194.09 | *Z. corallinum Hance* | [94] |
| | | | | *Z. officinale* | [86] |
| 323 | 2-gingerol | $C_{13}H_{18}O_4$ | 238.12 | *Z. officinale* | [115] |
| 324 | 4-gingerol | $C_{15}H_{22}O_4$ | 266.15 | *Z. officinale* | [115] |
| 325 | 6-gingerol | $C_{17}H_{26}O_4$ | 294.18 | *Z. officinale* | [115] |
| 326 | 8-gingerol | $C_{19}H_{30}O_4$ | 322.21 | *Z. officinale* | [115] |
| 327 | 10-gingerol | $C_{21}H_{34}O_4$ | 350.25 | *Z. officinale* | [115] |
| 328 | 12-gingerol | $C_{23}H_{38}O_4$ | 378.28 | *Z. officinale* | [115] |
| 329 | 6-gingerdiol | $C_{17}H_{28}O_4$ | 296.20 | *Z. officinale* | [115] |
| | | | | *Z. mioga* | [112] |
| 330 | 8-gingerdiol | $C_{19}H_{32}O_4$ | 324.23 | *Z. officinale* | [115] |
| 331 | 10-gingerdiol | $C_{21}H_{36}O_4$ | 352.26 | *Z. officinale* | [115] |
| 332 | 4-shogaol | $C_{15}H_{20}O_3$ | 248.14 | *Z. officinale* | [115] |
| 333 | 6-shogaol | $C_{17}H_{24}O_3$ | 276.17 | *Z. officinale* | [115] |
| 334 | 8-shogaol | $C_{19}H_{28}O_3$ | 304.20 | *Z. officinale* | [115] |
| 335 | 10-shogaol | $C_{21}H_{32}O_3$ | 332.24 | *Z. officinale* | [115] |
| 336 | 12-shogaol | $C_{23}H_{36}O_3$ | 360.27 | *Z. officinale* | [115] |
| 337 | Methyl-6-shogaol | $C_{18}H_{26}O_3$ | 290.19 | *Z. officinale* | [118] |
| 338 | 6-hydroxyl-6-shogaol | $C_{17}H_{24}O_4$ | 292.17 | *Z. officinale* | [115] |
| 339 | Methyl-6-gingerol | $C_{18}H_{28}O_4$ | 308.20 | *Z. officinale* | [115] |
| 340 | Methyl-8-gingerol | $C_{20}H_{32}O_4$ | 336.23 | *Z. officinale* | [115] |
| 341 | Methyl-10-gingerol | $C_{22}H_{36}O_4$ | 364.26 | *Z. officinale* | [115] |
| 342 | Methyl-6-gingerdiol | $C_{18}H_{30}O_4$ | 310.21 | *Z. officinale* | [115] |
| 343 | Acetoxy-8-gingerol | $C_{21}H_{32}O_5$ | 364.22 | *Z. officinale* | [115] |

| | | | | | |
|---|---|---|---|---|---|
| 344 | Acetoxy-10-gingerol | $C_{23}H_{36}O_5$ | 392.26 | *Z. officinale* | [115] |
| 345 | Methylacetoxy-6-gingerol | $C_{20}H_{30}O_5$ | 350.21 | *Z. officinale* | [115] |
| 346 | 3-Acetoxy-6-gingerdiol | $C_{19}H_{30}O_5$ | 338.21 | *Z. officinale* | [115] |
| 347 | 5-Acetoxy-7-gingerdiol | $C_{20}H_{32}O_5$ | 352.22 | *Z. officinale* | [118] |
| 348 | Methyl diacetoxy-4-gingerdiol | $C_{20}H_{30}O_6$ | 366.20 | *Z. officinale* | [118] |
| 349 | Methyl-3-acetoxy-6-gingerdiol | $C_{20}H_{32}O_5$ | 352.22 | *Z. officinale* | [115] |
| 350 | Methyldiacetoxy-6-gingerdiol | $C_{22}H_{34}O_6$ | 394.24 | *Z. officinale* | [115] |
| 351 | Diacetoxy-8-gingerdiol | $C_{23}H_{36}O_6$ | 408.25 | *Z. officinale* | [115] |
| 352 | 6-gingerdione | $C_{17}H_{24}O_4$ | 292.17 | *Z. officinale* | [119] |
| 353 | 8-gingerdione | $C_{19}H_{28}O_4$ | 320.20 | *Z. officinale* | [115] |
| 354 | 10-gingerdione | $C_{21}H_{32}O_4$ | 348.23 | *Z. officinale* | [115] |
| 355 | 6-paradol | $C_{17}H_{26}O_3$ | 278.19 | *Z. officinale* | [115] |
| 356 | 7-paradol | $C_{18}H_{29}O_3$ | 292.20 | *Z. officinale* | [118] |
| 357 | 6-dehydrogingerdione | $C_{17}H_{22}O_4$ | 290.15 | *Z. officinale* | [115] |
| 358 | 8-dehydrogingerdione | $C_{19}H_{26}O_4$ | 318.18 | *Z. officinale* | [115] |
| 359 | 10-dehydrogingerdione | $C_{21}H_{30}O_4$ | 346.21 | *Z. officinale* | [115] |
| 360 | 12-dehydrogingerdione | $C_{23}H_{34}O_4$ | 374.25 | *Z. officinale* | [115] |
| 361 | Geranjdferu licacid | $C_{20}H_{26}O_4$ | 330.18 | *Z. officinale* | [119] |
| 362 | 8-isodehydro gingerdione | $C_{19}H_{26}O_4$ | 318.18 | *Z. officinale* | [119] |
| 363 | 6-isodehydro gingerdione | $C_{17}H_{22}O_4$ | 290.15 | *Z. officinale* | [119] |
| 364 | 1-(4-hydroxy-3-methoxyphenyl)-3-tetrade canone | $C_{21}H_{34}O_3$ | 334.25 | *Z. officinale* | [119] |
| 365 | 6-gingesulfonic acid | $C_{17}H_{26}O_6S$ | 358.15 | *Z. officinale* | [119] |
| 366 | 8-gingesulfonic acid | $C_{19}H_{30}O_6S$ | 386.18 | *Z. officinale* | [119] |
| 367 | 10-gingesulfonic acid | $C_{21}H_{34}O_6S$ | 414.21 | *Z. officinale* | [119] |
| | ***Organic acids*** | | | | |
| 368 | 4-hydroxybenzoic acid | $C_7H_6O_3$ | 138.03 | *Z. striolatum Diels* | [121] |
| 369 | 3-hydroxy-4-methoxybenzoic acid | $C_8H_8O_4$ | 168.04 | *Z. striolatum Diels* | [121] |
| 370 | Linoleic acid | $C_{17}H_{30}O_3$ | 282.22 | *Z. striolatum Diels* | [23] |
| | | | | *Z. officinale* | [86] |
| 371 | (E)-16-methylheptadec-2-enoic acid | $C_{18}H_{34}O_2$ | 282.26 | *Z. striolatum Diels* | [122] |
| | | | | *Z. officinale* | [23] |
| 372 | $\alpha$-linolenic acid | $C_{18}H_{30}O_2$ | 278.22 | *Z. officinale* | [86] |
| | | | | *Z. mioga* | [112] |
| 373 | Glycosmisic acid | $C_{20}H_{20}O_7$ | 372.12 | *Z. officinale* | [99] |
| 374 | (3,4-Dimethoxyphenyl) Acetic Acid | $C_{10}H_{12}O_4$ | 196.07 | *Z. officinale* | [99] |
| 375 | (2Z,6Z)-2,6-dimethylocta-2,6-dienedioic acid | $C_{10}H_{14}O_4$ | 198.09 | *Z. officinale* | [99] |
| 376 | Isovaleric acid | $C_5H_{10}O_2$ | 102.07 | *Z. officinale* | [88] |
| 377 | Hexanoic acid | $C_6H_{11}O_2$ | 116.08 | *Z. officinale* | [88] |
| 378 | Tetracosanoic acid | $C_{24}H_{48}O_2$ | 368.37 | *Z. officinale* | [117] |
| 379 | Melissic acid | $C_{30}H_{60}O_2$ | 452.46 | *Z. officinale* | [117] |
| 380 | 9,12-octadecadienoic acid | $C_{18}H_{32}O_2$ | 280.24 | *Z. officinale* | [60] |
| 381 | 3-acetamidobenzoic acid | $C_9H_9NO_3$ | 179.06 | *Z. officinale* | [60] |
| 382 | Dihydrophaseic acid | $C_{13}H_{18}O_6$ | 270.11 | *Z. officinale* | [123] |
| 383 | 4-hydroxy-3,5-dimethoxybenzoic acid | $C_9H_{10}O_5$ | 198.05 | *Z. officinale* | [119] |
| 384 | 3-(4-hydroxy-3-methoxyphenyl)propanoic acid | $C_{10}H_{12}O_4$ | 196.07 | *Z. officinale* | [119] |
| 385 | p-hydroxybenzene propanoic acid | $C_9H_{10}O_3$ | 166.06 | *Z. officinale* | [119] |

| | | | | | |
|---|---|---|---|---|---|
| 386 | Pinocarveyl acetate trans- | $C_{12}H_{18}O_2$ | 194.13 | *Z. mioga* | [82] |
| 387 | Propanoic acid | $C_3H_6O_2$ | 74.04 | *Z. mioga* | [112] |
| 388 | Glutaric acid | $C_5H_8O_4$ | 132.04 | *Z. mioga* | [112] |
| 389 | Ethylmalonic acid | $C_4H_6O_4$ | 118.03 | *Z. mioga* | [112] |
| 390 | Citric acid | $C_7H_{10}O_7$ | 206.04 | *Z. mioga* | [112] |
| 391 | Phenylalanine | $C_9H_{11}NO_2$ | 165.08 | *Z. mioga* | [112] |
| 392 | 2-(Methylamino)benzoic acid | $C_8H_9NO_2$ | 151.06 | *Z. mioga* | [112] |
| 393 | Tryptophan | $C_{11}H_{12}N_2O_2$ | 204.09 | *Z. mioga* | [112] |
| 394 | Oxalic acid | $C_2H_2O_4$ | 90.03 | *Z. officinale* | [30] |
| 395 | Tartaric acids | $C_4H_6O_6$ | 150.02 | *Z. officinale* | [30] |
| | | ***Sterides*** | | | |
| 396 | Daucosterol | $C_{35}H_{60}O_6$ | 576.44 | *Z. corallinum Hance* | [94] |
| | | | | *Z. striolatum Diels* | [124] |
| | | | | *Z. officinale* | [118] |
| 397 | β-sitosterol | $C_{30}H_{52}O$ | 428.40 | *Z. striolatum Diels* | [124] |
| | | | | *Z. officinale* | [118] |
| | | ***Others*** | | | |
| 398 | 4-(4-hydroxy-3-methoxyphenyl)but-3-en-2-one | $C_{11}H_{12}O_3$ | 192.08 | *Z. officinale* | [100] |
| 399 | 1-(4-hydroxy-3-methoxyphenyl)ethanone | $C_9H_{10}O_3$ | 166.06 | *Z. officinale* | [100] |
| 400 | 2,5-dibutylfuran | $C_{12}H_{20}O$ | 180.15 | *Z. officinale* | [60] |
| 401 | Tetrahydrofuran | $C_4H_8O$ | 72.06 | *Z. mioga* | [82] |
| 402 | Glycerol | $C_3H_8O_3$ | 92.05 | *Z. mioga* | [112] |
| 403 | Hedycaryol | $C_{15}H_{26}O$ | 222.20 | *Z. zerumbet* | [7] |
| 404 | p-hydroxybenzaldehyde | $C_7H_6O_2$ | 122.04 | *Z. zerumbet* | [114] |
| | | | | *Z. officinale* | [125] |
| 405 | Vanillin | $C_8H_8O_3$ | 152.05 | *Z. zerumbet* | [114] |
| | | | | *Z. corallinum Hance* | [83] |
| 406 | Zerumbone epoxide | $C_{16}H_{24}O_2$ | 348.18 | *Z. zerumbet* | [126] |
| 407 | Sucrose | $C_{12}H_{22}O_{11}$ | 334.12 | *Z. striolatum Diels* | [124] |
| 408 | α-tritiated thymidine | $C_5H_5TN_2O_2$ | 128.05 | *Z. striolatum Diels* | [121] |
| 409 | Uracil | $C_4H_4N_2O_2$ | 112.03 | *Z. striolatum Diels* | [121] |
| | | | | *Z. officinale* | [117] |
| 410 | Hexanal | $C_6H_{12}O$ | 100.09 | *Z. officinale* | [86] |
| 411 | Decane-2-dione | $C_{10}H_{20}O$ | 156.15 | *Z. officinale* | [86] |
| 412 | Undecanal | $C_{11}H_{22}O$ | 170.17 | *Z. officinale* | [86] |
| 413 | 3-undecyne | $C_{11}H_{2}0$ | 152.16 | *Z. mioga* | [82] |
| 414 | 3,7,11,15-tetramethylhexadeca-1,3,6,10,14-pentaene | $C_{20}H_{32}$ | 272.25 | *Z. officinale* | [86] |
| 415 | 3,3,6-trimethylhepta-4,5-diene-2-one | $C_{10}H_{16}O$ | 152.12 | *Z. officinale* | [86] |
| 416 | Citrylidenmalonsaeure | $C_{12}H_{16}O_4$ | 224.10 | *Z. officinale* | [41] |
| 417 | 3-oxabicyclo[3.2.0]heptane-2,4-dione | $C_6H_6O_3$ | 126.03 | *Z. officinale* | [118] |
| 418 | Indole-3-carboxaldehyde | $C_9H_7NO$ | 145.05 | *Z. officinale* | [99] |

| | | | | | |
|---|---|---|---|---|---|
| 419 | (Z)-2,6-dimethylocta-6-diene-1,8-diol | $C_{10}H_{20}O_2$ | 172.15 | *Z. officinale* | [99] |
| 420 | Acetal | $C_6H_{14}O_2$ | 118.10 | *Z. officinale* | [88] |
| 421 | Heptan-2-one | $C_7H_{14}O$ | 114.10 | *Z. officinale* | [88] |
| 422 | Heptaldehyde | $C_7H_{14}O$ | 114.10 | *Z. officinale* | [88] |
| 423 | Valeraldehyde propyleneglycol acetal 2 | $C_8H_{16}O_2$ | 144.12 | *Z. officinale* | [88] |
| 424 | trans-2-heptenal | $C_7H_{12}O$ | 112.09 | *Z. officinale* | [88] |
| 425 | 2-ethylhexanol | $C_8H_{18}O$ | 130.14 | *Z. officinale* | [88] |
| 426 | 2-nonanol | $C_9H_{20}O$ | 144.15 | *Z. officinale* | [88] |
| 427 | trans-2-decenal | $C_{10}H_{18}O$ | 154.14 | *Z. officinale* | [88] |
| 428 | 2-undecanol | $C_{11}H_{24}O$ | 172.18 | *Z. officinale* | [88] |
| 429 | (+)-isolariciresinol | $C_{20}H_{24}O_6$ | 360.16 | *Z. officinale* | [100] |
| 430 | Ethyl 4'-hydroxy-3'-methoxycinnamate | $C_{12}H_{14}O_4$ | 222.09 | *Z. officinale* | [100] |
| 431 | (+)-pinoresinol | $C_{20}H_{22}O_6$ | 358.14 | *Z. officinale* | [100] |
| 432 | 1-octen-3-o1 | $C_8H_{16}O$ | 128.12 | *Z. mioga* | [82] |
| 433 | bis(2-ethylOctyl) phthalate | $C_{28}H_{46}O_4$ | 446.34 | *Z. officinale* | [117] |
| 434 | 5-hydroxymethylfurfural | $C_6H_6O_3$ | 126.03 | *Z. officinale* | [117] |
| 435 | Isovanillin | $C_8H_8O_3$ | 152.05 | *Z. officinale* | [125] |
| 436 | Carinol | $C_{20}H_{26}O_7$ | 378.17 | *Z. officinale* | [60] |
| 437 | 1,4-naphthoquinon | $C_{10}H_6O_2$ | 158.04 | *Z. officinale* | [60] |
| 438 | 3,5,6-trihydroxy-7-megastigmen-9-one | $C_{13}H_{22}O_4$ | 242.15 | *Z. officinale* | [123] |
| 439 | 3-(3-hydroxybutyl)-2,4,4-trimethyl-2,5-cyclohexadien-1-one | $C_{13}H_{20}O_2$ | 208.15 | *Z. officinale* | [123] |
| 440 | Grasshopper ketone | $C_{13}H_{20}O_3$ | 224.14 | *Z. officinale* | [123] |
| 441 | 1,3,6-tri-hydroxy-8-methyl-anthra-quinone | $C_{15}H_{10}O_5$ | 270.05 | *Z. officinale* | [119] |
| 442 | 3′,5-diallylbiphenyl-2,4′-diol | $C_{18}H_{18}O_2$ | 266.13 | *Z. officinale* | [119] |
| 443 | Butylbenzene | $C_{10}H_{14}$ | 134.11 | *Z. mioga* | [82] |
| 444 | 2-methylbutanal | $C_5H_{10}O$ | 86.07 | *Z. mioga* | [82] |
| 445 | 3-hydroxy-2-butanone | $C_4H_8O_2$ | 88.05 | *Z. mioga* | [82] |
| 446 | 2-acetylcyclopentanone | $C_7H_{10}O_2$ | 126.07 | *Z. mioga* | [82] |
| 447 | D-Mannose | $C_6H_{12}O_6$ | 180.06 | *Z. mioga* | [112] |

**Table S2.** Antimicrobial effect of *Zingiber* plants.

| Extracts/Compounds | Microbes | Inhibitory concentrations (μg/mL) | | References |
|---|---|---|---|---|
| 10-gingerol | *P. gingivalis ATCC 53978* | MIC: 6 | MBC: 6 | [55] |
| | *Porphyromonas endodontalis ATCC 35406* | MIC: 8 | MBC: 4 | |
| | *P. intermedia ATCC 25611* | MIC: 14 | MBC: 14 | |
| | *Mycobacterium avium* | MIC: 25 | | |
| | *Mycobacterium tuberculosis* | MIC: 50 | | |
| 12-gingerol | *P. gingivalis ATCC 53978* | MIC: 15 | MBC: 10 | |
| | *Porphyromonas endodontalis ATCC 35406* | MIC: 15 | MBC: 10 | |
| | *P. intermedia ATCC 25611* | MIC: 30 | MBC: 20 | |
| 6-gingerol | *Helicobacter pylori* | MIC: 20 | | [127] |
| Zerumbone | *S. mutans ATCC35668* | MIC: 250 | MBC: 500 | [54] |
| | *E. faecalis ATCC 29212* | MIC: 250 | MBC: 250 | [39] |
| | *E. faecalis ATCC 29212* | MIC: 250 | MBC: 250 | |
| | *E. faecalis ATCC 29212* | MIC: 250 | MBC: 250 | |
| | *E. faecalis ATCC 29212* | MIC: 250 | MBC: 250 | |
| | *E. faecalis ATCC 29212* | MIC: 250 | MBC: 250 | |
| | *E. faecalis ATCC 29212* | MIC: 250 | MBC: 250 | |
| | *E. faecalis ATCC 29212* | MIC: 250 | MBC: 250 | |
| | *H. pylori NCTC11637* | MIC: 250 | | [54] |
| | *H. pylori J99* | MIC: 250 | | |
| | *C. albicans ATCC 14053* | MIC: 64 | | [53] |
| | *E. floccosum* | MIC: 8 | MFC: 64 | [128] |
| | *M. canis* | MIC: 8 | MFC: 64 | |
| | *M. gypseum* | MIC: 16 | MFC: 128 | |
| | *T. rubrum* | MIC: 16 | MFC: 128 | |
| *Z. zerumbet* fresh rhizome essential oil | *E. faecalis ATCC 29212* | MIC: 1250 | MBC: 1250 | |
| | *S. aureus ATCC 6538P* | MIC: 78.13 | MBC: 156.25 | |
| | *B. subtilis CMCC (B) 63501* | MIC: 78.13 | MBC: 156.25 | |
| | *P. aeruginosa CMCC (B)10104* | MIC: 312.5 | MBC: 625.00 | |
| | *E. coli ATCC 25922* | MIC: 156.25 | MBC: 312.25 | |
| | *P. vulgaris CMCC (B) 49027* | MIC: 78.13 | MBC: 156.25 | |
| | *C. albicans CMCC (F) 98001* | MIC: 312.5 | MBC: 2500.0 | |
| *Z. zerumbet* dry rhizome essential oil | *E. faecalis ATCC 29212* | MIC: 1250 | MBC: 1250 | |
| | *S. aureus ATCC 6538P* | MIC: 156.25 | MBC: 156.25 | |
| | *B. subtilis CMCC (B) 63501* | MIC: 156.25 | MBC: 312.50 | |
| | *P. aeruginosa CMCC (B)10104* | MIC: 312.50 | MBC: 625.00 | |
| | *E. coli ATCC 25922* | MIC: 156.25 | MBC: 625.00 | |
| | *P. vulgaris CMCC (B) 49027* | MIC: 156.25 | MBC: 312.50 | |
| | *C. albicans CMCC (F) 98001* | MIC: 312.50 | MBC: 2500.00 | |
| | *Acinetobacter baumannii* | MIC: 1457.81 | MBC: 1457.81 | [129] |
| *Z. corallinum* essential oil | *Rhizoctonia solani Kühn* | $EC_{50}$: 3.64 | | [95] |
| | *Fusarium gram inearum* | $EC_{50}$: 11.20 | | |
| | *Fusarium nivale* | $EC_{50}$: 11.33 | | |
| | *Pyricularia oryzae* | $EC_{50}$: 14.19 | | |
| | *Trichophyton rubrum* | MIC: 0.3 | | [130] |
| | *Trichophyton Mentagrophytes* | MIC: 0.5 | | |
| | *Trichophyton Rosaceum* | MIC: 0.5 | | |
| | *Trichoderma hirsutum* | MIC: 1 | | |
| | *Trichoderma flocculus* | MIC: 0.5 | | |
| | *Microsporum gypseum* | MIC: 1 | | |
| | *Microsporum ferrugineum* | MIC: 1 | | |
| | *Microsporum lanosum* | MIC: 1 | | |

| | | | |
|---|---|---|---|
| | *Candida Albicans* | MIC: 5 | |
| | *Cryptoccus neo formans* | MIC: 2 | |
| | *Fonsecaea pedrosoi* | MIC: 2 | |
| | *Sporothrix schenckii* | MIC: 2 | |
| Miogadial | *Bacillus cereus ATCC 10702* | MIC: 25 | [25] |
| | *Bacillus subtilis ATCC 6633* | MIC: 25 | |
| | *Staphlococcus aureus ATCC 12600* | MIC: 60 | |
| | *Staphlococcus aureus ATCC 361* | MIC: 25 | |
| | *Staphlococcus epidermidis ATCC 14990* | MIC: 25 | |
| | *Staphlococcus faecalis RIMD 3116001* | MIC: 125 | |
| | *Candida albicans NBRC 2091* | MIC: 50 | |
| | *Candida tropicalis NBRC 66029* | MIC: 50 | |
| | *Candida glabata NBRC 2001* | MIC: 100 | |
| | *Zygosaccharomyces rouxii NBRC 28253* | MIC: 50 | |
| | *Saccharomyces cerevisiae NBRC 2601* | MIC: 50 | |
| | *Aspergillus fumigatus NBRC 7080* | MIC: 500 | |
| | *Penicillium frequentans NBRC 7919* | MIC: 500 | |
| Galanal A | *Bacillus cereus ATCC 10702* | MIC: 200 | |
| | *Bacillus subtilis ATCC 6633* | MIC: 500 | |
| | *Staphlococcus aureus ATCC 12600* | MIC: 500 | |
| | *Staphlococcus aureus ATCC 361* | MIC: 500 | |
| | *Staphlococcus epidermidis ATCC 14990* | MIC: 500 | |
| | *Candida albicans NBRC 2091* | MIC: 200 | |
| | *Candida tropicalis NBRC 66029* | MIC: 200 | |
| | *Candida glabata NBRC 2001* | MIC: 500 | |
| | *Zygosaccharomyces rouxii NBRC 28253* | MIC: 200 | |
| | *Saccharomyces cerevisiae NBRC 2601* | MIC: 200 | |
| Galanal B | *Bacillus cereus ATCC 10702* | MIC: 200 | |
| | *Bacillus subtilis ATCC 6633* | MIC: 300 | |
| | *Staphlococcus aureus ATCC 12600* | MIC: 500 | |
| | *Staphlococcus aureus ATCC 361* | MIC: 300 | |
| | *Staphlococcus epidermidis ATCC 14990* | MIC: 300 | |
| | *Candida albicans NBRC 2091* | MIC: 200 | |
| | *Candida tropicalis NBRC 66029* | MIC: 200 | |
| | *Candida glabata NBRC 2001* | MIC: 500 | |
| | *Zygosaccharomyces rouxii NBRC 28253* | MIC: 200 | |
| | *Saccharomyces cerevisiae NBRC 2601* | MIC: 200 | |

MFC: minimum fungicidal concentration; MIC: minimum inhibitory concentration; MBC: minimum bactericidal concentration; $EC_{50}$: the half maximal effective concentration.

**Table S3.** Larvicidal effect of *Zingiber* plants.

| Extracts/Compounds | Insects | Inhibitory concentrations (μg/mL) | | References |
|---|---|---|---|---|
| Methanol extract of *Z. zerumbet* rhizomes | *Artemia salina* | 24h: $LC_{50}$ = 127.4 | $LC_{90}$ = 351.4 | [57] |
| | | 48h: $LC_{50}$ = 64.0 | $LC_{90}$ = 195.6 | |
| | *Aedes aegypti* | 48h: $LC_{50}$ = 293.2 | $LC_{90}$ = 471.6 | |
| | | 72h: $LC_{50}$ = 232.3 | $LC_{90}$ = 382.6 | |
| | *Anopheles nuneztovari* | 48h: $LC_{50}$ = 73.9 | $LC_{90}$ = 227.9 | |
| | | 72h: $LC_{50}$ = 68.1 | $LC_{90}$ = 210.5 | |
| Dichloromethane extract of *Z. zerumbet* rhizomes | *Aedes aegypti* | 24h: $LC_{50}$ = 40.4 | $LC_{90}$ = 177.7 | |
| | | 48h: $LC_{50}$ = 30.9 | $LC_{90}$ = 132.8 | |
| | *Artemia salina* | 48h: $LC_{50}$ = 89.8 | $LC_{90}$ = 170.5 | |
| | | 72h: $LC_{50}$ = 68.2 | $LC_{90}$ = 141.4 | |
| | *Anopheles nuneztovari* | 48h: $LC_{50}$ = 62.8 | $LC_{90}$ = 206.4 | |
| | | 72h: $LC_{50}$ = 54.6 | $LC_{90}$ = 142.8 | |
| *Z. corallinum* essential oil | *Aedes albopictus* | 24h: $LC_{50}$ = 77.994 | | [56] |
| | *Culex quinquefasciatus* | 24h: $LC_{50}$ = 66.574 | | |

$LC_{50}$: lethal concentration 50%; $LC_{90}$: lethal concentration 90%.
